# Experimental evolution reveals genetic and transcriptomic mechanisms underlying benzalkonium chloride tolerance and fluoroquinolone cross-resistance in *Listeria monocytogenes*

**DOI:** 10.1128/msystems.01809-25

**Published:** 2026-07-24

**Authors:** Tyler D. Bechtel, Julia Hershelman, Adenike Adeyanju, David C. Rinker, Mrinalini Ghoshal, Lynne A. McLandsborough, John G. Gibbons

**Affiliations:** 1 Department of Food Science, University of Massachusetts 504174, Amherst, Massachusetts, USA; 2 Department of Biological Sciences, Vanderbilt University 5718, Nashville, Tennessee, USA; 3 Department of Microbiology, University of Massachusetts 196202, Amherst, Massachusetts, USA; 4 Molecular and Cellular Biology Graduate Program, University of Massachusetts 14707, Amherst, Massachusetts, USA; 5 Organismic & Evolutionary Biology Graduate Program, University of Massachusetts 14707, Amherst, Massachusetts, USA; University of California Irvine, Irvine, California, USA

**Keywords:** *Listeria monocytogenes*, experimental evolution, benzalkonium chloride, efflux-mediated tolerance, phosphotransferase system, antibiotic cross-resistance

## Abstract

Preventing *Listeria monocytogenes* persistence in food processing environments is challenged by tolerance to quaternary ammonium compound sanitizers such as benzalkonium chloride (BAC). To define the genetic and molecular routes to BAC tolerance and antibiotic cross-resistance, we performed an evolve-and-resequence experiment on three *L. monocytogenes* strains (three replicate lineages each) passaged under sublethal BAC for ~400–500 generations. All nine BAC-exposed lineages increased BAC minimum inhibitory concentrations (MICs), and several showed elevated tolerance to a commercial sanitizer. Notably, multiple lineages displayed cross-resistance to fluoroquinolones. Whole-genome sequencing of end-point lineages revealed recurrent, parallel mutations in the *fepR* transcriptional regulator (often involving nonsense mutations), accompanied by missense mutations in the adjacent efflux pump *fepA*, implicating derepressed multidrug and toxic compound extrusion (MATE) efflux activity in BAC, and fluoroquinolone tolerance. RNA-seq under BAC stress showed consistent upregulation of phosphotransferase system (PTS) genes across evolved lineages. Complementing this, we observed convergent missense mutations in *ptsH* (encoding the HPr phosphocarrier), including substitutions in the HPr serine phosphorylation region. Biophysical modeling predicted that these HPr variants are strongly destabilizing, consistent with altered PTS signaling under BAC sthe presence of BAC stress tress. Efflux inhibition with reserpine reduced BAC tolerance in *fepR*/*fepA*-mutant backgrounds, whereas the effects were minimal in a bcrABC-positive strain (where *bcrABC* encodes a plasmid-borne BAC resistance efflux cassette), underscoring strain-dependent mechanisms. Together, our data reveal efflux-driven and PTS-linked adaptation to BAC with implications for the emergence of antibiotic cross-resistance and persistence in food production environments.

**IMPORTANCE:**

*Listeria monocytogenes* persistence in food facilities poses major public health and economic burdens. We modeled long-term exposure to sublethal benzalkonium chloride (BAC) to approximate conditions similar to those in food processing facility drains and uncovered two complementary routes to sanitizer tolerance: (i) repeated inactivation of the fepR regulator with accompanying alterations in the fepA efflux pump, driving BAC tolerance and fluoroquinolone cross-resistance, and (ii) convergence on mutations in ptsH (HPr) within the phosphotransferase system, supported by RNA-seq upregulation of phosphotransferase system (PTS) pathways and biophysical evidence of destabilized HPr variants. Efflux inhibition reduced BAC tolerance where fepR/fepA was mutated but had limited impact in a bcrABC-positive background (where bcrABC encodes a plasmid-borne BAC resistance efflux cassette), highlighting the role of genetic context. These findings connect sanitizer adaptation to antibiotic resistance and identify PTS signaling as a systems-level contributor, informing surveillance and sanitation strategies in food production.

## INTRODUCTION

*Listeria monocytogenes* is a gram-positive, facultative anaerobe and foodborne pathogen that is the causative agent of a serious disease called listeriosis (1). Listeriosis is a rare but potentially fatal infection, accounting for less than 1% of all foodborne illnesses, but almost 28% of all foodborne illness-related deaths (2). Due to its high mortality rate, it is important to understand how listeriosis is transmitted to humans through food. The first factor that contributes to *L. monocytogenes* transmission into food is the ubiquitous nature of this bacterium in the natural environment. *L. monocytogenes* occurs naturally in a diversity of ecological niches (including soil, vegetation, water, and animals), which presents many possible entry points for contamination during the food manufacturing process (3). The prevalence of this pathogen makes it extremely difficult to prevent its introduction into the food processing environment (FPE). Within the FPE, various biocides and stressors are utilized during the food manufacturing process to prevent microbial contamination. However, *L. monocytogenes* is naturally stress tolerant and is capable of withstanding many of these stressors, including refrigeration temperatures (0°C–4°C), a wide pH range (4.6–9.6), desiccation, and high salt conditions (up to 10% [wt/vol] NaCl) (4–8). This resilience to extreme conditions allows *L. monocytogenes* to persist in the FPE, which presents a serious challenge for food manufacturers.

Persistence in the context of food safety is defined as repeated isolation of a single bacterial strain from the same food processing facility or location over an extended period of time (9). The issue of persistence is compounded by the ability of *L. monocytogenes* to form biofilms, which provides mechanical strength to its adherence to surfaces and enables the bacterium to colonize food contact surfaces over long periods of time (10, 11). Furthermore, *L. monocytogenes* biofilms are known to provide increased resistance to antimicrobials (12). Together, these characteristics exemplify the threat that bacterial persistence presents, which could have severe downstream implications for both human health and economic losses. For instance, it is estimated that *L. monocytogenes* causes a $2.8 billion economic burden in the United States annually, most of which is due to costs associated with deaths ($2.1 billion) (13).

Quaternary ammonium compounds (QACs) are a group of chemical sanitizers frequently used in the food industry (9, 14). QACs consist of a central ammonium group that carries a permanent positive charge that is commonly bonded to alkyl and aromatic substituents (15). The properties and performance of QACs are influenced by the nature of the substituents attached and the length of the alkyl chain; however, all QACs function by disrupting the cellular membrane, causing leakage of cellular components and dysfunctional membrane permeability (14). Benzalkonium chloride (BAC), the focus of the present study, is one of the most commonly used QACs (16). Unfortunately, several studies have shown multiple incidences of *L. monocytogenes* strains that are associated with foodborne outbreaks exhibiting increased BAC tolerance (14, 17, 18). For example, *L. monocytogenes* strain H7550, the causative agent of a deadly 1998–1999 listeriosis outbreak, exhibited high BAC tolerance with an MIC of 40 μg/mL (which greatly exceeds the MIC breakpoint of 4 μg/mL that is generally considered BAC-resistant) (18, 19). Outbreaks such as this highlight the importance of understanding how tolerance to BAC evolves and the genetic determinants underlying increased BAC tolerance in *L. monocytogenes*.

The majority of mechanisms underlying QAC tolerance in *L. monocytogenes* center on efflux. A number of different efflux pumps are associated with decreased susceptibility to QAC sanitizers (20). For instance, *bcrABC* is a BAC resistance cassette that is plasmid-borne or mobile element-associated and is perhaps the most prevalent of the various *L. monocytogenes* QAC efflux genotypes (21, 22). Due to its association with food products and recurrent isolation in the FPE, the *bcrABC* cassette has been identified as a potential biomarker for persistence in *L. monocytogenes*. Another QAC-tolerant genotype that is prevalent among *L. monocytogenes* food isolates is the efflux transporter QacH and its transcriptional regulator TetR, which is known to export QACs from within the cell using the proton motive force (22, 23). The multidrug toxic compound extrusion (MATE) efflux pump, FepA, has also been identified as a key genetic target for adaptation to QACs (24). There is evidence of mutations in the *fepAR* operon conferring increased tolerance to QACs and off-target resistance to antibiotics (25). These mechanisms are just a few examples demonstrating the prevailing consensus in the literature that bacterial efflux pumps are the primary determinant of QAC sanitizer tolerance in *L. monocytogenes*.

The evolve and resequence approach (E&R) combines adaptive laboratory evolution (ALE) with whole-genome sequencing and offers a powerful tool to examine the genetic and molecular mechanisms underlying phenotypic evolution (26–29). In this approach, bacterial isolates are cultured for a prolonged period in a controlled environment and allowed to adapt to the novel environment. Using the E&R strategy, several studies have investigated the genetic basis of antimicrobial resistance and BAC tolerance among persistent *L. monocytogenes* strains after short-term exposure to sublethal concentrations of BAC (19, 30, 31). Bolten et al. (30) demonstrated that after serial passage of *L. monocytogenes* in incrementally increasing concentrations of BAC, 62 of 67 strains exhibited higher MICs than the parent strains. Genomic analysis of these strains identified nonsynonymous mutations of the *fepR* transcriptional regulator in the majority of BAC-adapted strains. To et al. (19) and Romanova et al. (31) both passaged *L. monocytogenes* in progressively increasing concentrations of BAC over a 2- to 3-week period, and each found that efflux pump activity was the major mechanism of adaptation among BAC-sensitive strains, but that efflux pump activity only played a minimal role in adaptation among BAC-tolerant strains (19, 31). While these studies established *fepR/fepA*-mediated efflux as a primary mechanism of BAC adaptation, they were limited in evolutionary timescale, strain diversity, and mechanistic depth.

In food processing environments, surface cleaning can result in diluted sanitizers entering floor drains where they may encounter foodborne pathogens, and this prolonged exposure to sublethal sanitizer concentrations could drive bacterial adaptation. To model this scenario, we applied the E&R approach to three phylogenetically diverse *L. monocytogenes* strains passaged for ~400–500 generations under sublethal BAC; a timescale substantially longer than prior studies and more representative of chronic environmental exposure. Two strains belong to Lineage II serotype 1/2a and are closely related but differ in the presence of the *bcrABC* benzalkonium chloride resistance cassette, enabling direct comparison of how this persistence biomarker shapes evolutionary outcomes. A third strain, a serotype 4c Lineage IV isolate from a goat listeriosis outbreak, was included to assess whether adaptive mechanisms generalize across phylogenetically divergent backgrounds and because livestock-associated strains represent important entry points for *L. monocytogenes* contamination in the food chain. By integrating whole-genome sequencing, deep population sequencing, RNA-seq under BAC stress, and biophysical modeling, we provide a multi-layered mechanistic portrait of BAC adaptation (including the novel identification of *ptsH* and the PTS as an efflux-independent convergent target), which has not been achievable in previous studies.

## MATERIALS AND METHODS

### Adaptive laboratory evolution

Overnight cultures of ALE_10-WT, ALE_16-WT, and ALE_20-WT were used to establish three experimentally evolved lineages in TSB + YE and BAC (treatment), and three experimentally evolved lineages in TSB + YE (negative control) (a total of 18 evolved lineages). TSB + YE-evolved (BAC-) lineages were sequenced in parallel with BAC-evolved lineages to distinguish mutations driven by BAC selection from those arising due to adaptation to the growth medium or random drift. Specific mutations and genes identified in both BAC-evolved and TSB + YE-evolved lineages were considered likely media-adaptation artifacts and excluded from further analysis as candidates for BAC-specific tolerance. This conservative filtering approach increases confidence that the candidate genes highlighted in this study represent genuine targets of BAC-mediated selection. For the BAC treatments, the BAC concentration used for passaging was fixed at 1 μg/mL below the MIC of the respective ancestral strain and was not adjusted during the experiment. Strains were passaged into fresh media each day after 24 h growth at 37°C. Before each passage, the cell density was measured to maintain a consistent inoculum size. We inferred the number of generations as the cumulative number of cell divisions as previously described (32). Briefly, to estimate the number of cells in each culture, we first determined the CFU/mL at a normalized OD_600_ of 1.0. This value was then used to estimate the number of cells (*N*) at a given time by measuring optical density in each overnight culture as well as the number of cells transferred to fresh media each day. The dilution factor was adjusted with each passage according to the estimated cell count. The number of generations (*n*) was then calculated using the equation $n=\frac{log(N/N_{0})}{log(2)}$, where *N_0_* and *N* are the initial and final number of cells in a known volume, respectively. Each passage typically contained between ~6.0 × 10^6^ and 2.0 × 10^7^ cells.

**Fig 1:**
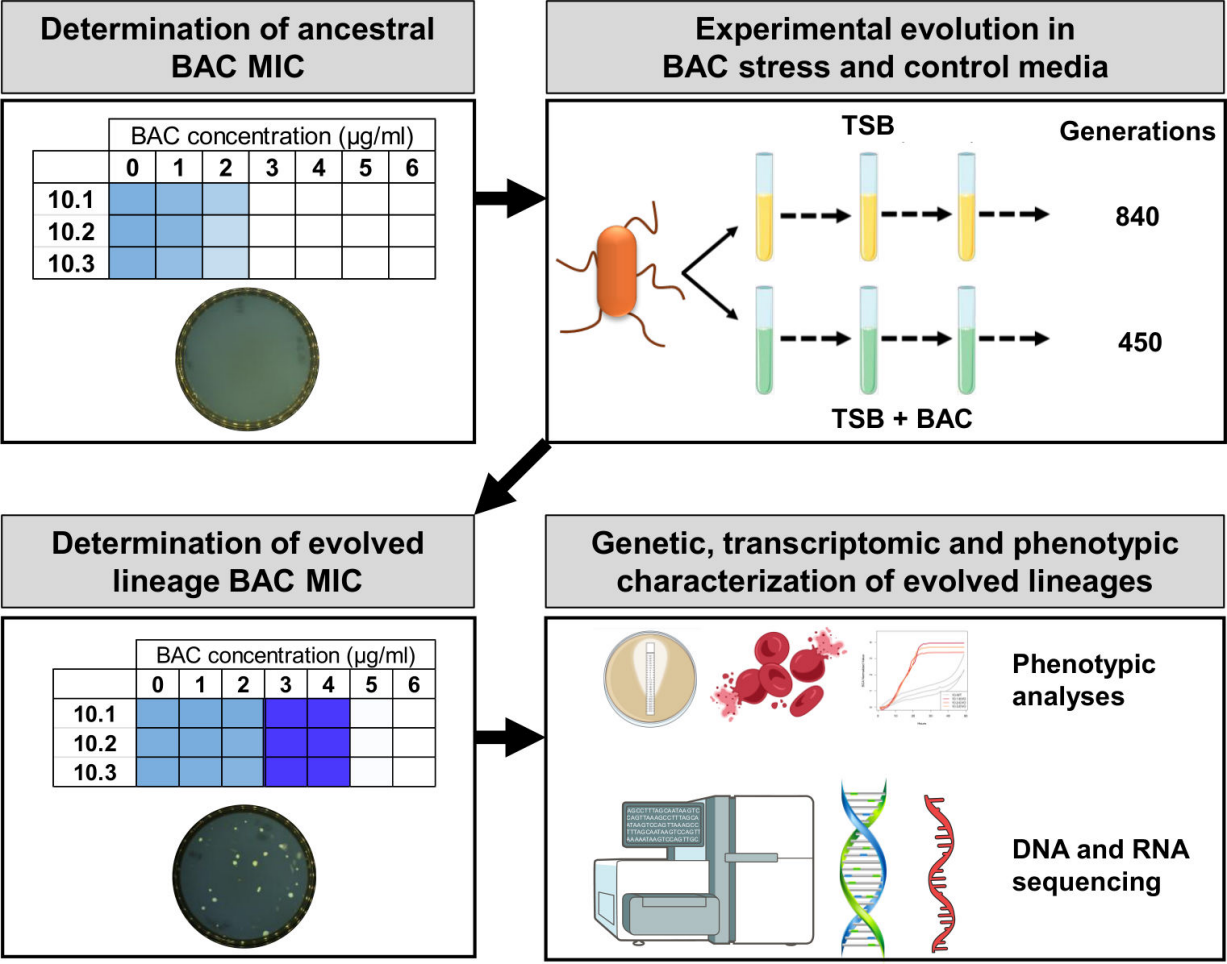
“Evolve and resequence” experimental design to model BAC tolerance in *L. monocytogenes*. The MIC of BAC was first measured for three phylogenetically distinct *L. monocytogenes* strains (ALE_10-*WT*, ALE_16-*WT*, and ALE_20-*WT*) with complete genome assemblies and annotations. Triplicates of each strain were then serially passaged in media containing sublethal concentrations of BAC (1 μg/mL below the MIC) for ~70 days. The MIC was reassessed for BAC-evolved strains (notated as ALE_10-*bac*, ALE_16-*bac*, and ALE_20-*bac*). The genomes of each BAC-evolved lineage were sequenced to identify mutations underlying increased BAC tolerance, and the transcriptomes of a subset of lineages were profiled and compared to their ancestors during BAC stress.

The experimental design is depicted in Fig. 1. The BAC MIC was measured intermittently (approximately every 10 days), at which point freezer stocks were prepared in 25% glycerol. Each isolate was passaged in both control (TSB + YE) and sanitizer-supplemented media (TSB + YE with BAC) for ~70 days. Throughout the course of the serial passaging experiment, freezer stocks were prepared from population-level cultures (i.e., the full overnight culture was archived without colony isolation). At the end-point of the experiment, both single-colony isolates and population samples were archived for each of the 18 lineages. Single colonies were used for whole-genome sequencing of evolved lineages, while population-level samples were used for deep sequencing to assess allele frequencies (see “Whole-genome sequencing of ancestral strains and evolved lineages” below).

### Bacterial isolates and culture conditions

All isolates used in this study were obtained from the Cornell University Food Safety Laboratory *Listeria* strains collection. Isolates FSL-N1-304 and FSL-N1-334 were both isolated from floor drains in a fish processing facility in New York, USA (33). FSL-J1-158 was isolated from a vaginal swab of an infected goat from a listeriosis outbreak in Georgia, USA (34, 35). For this study, isolates FSL-N1-304, FSL-N1-334, and FSL-J1-158 were renamed ALE_10-WT, ALE_16-WT, and ALE_20-WT, respectively (ALE = Adaptive Laboratory Evolution). All three strains were initially cultured, in triplicate, at 37°C in tryptic soy broth media supplemented with 0.6% yeast extract (TSB + YE). Frozen culture stocks were maintained in 25% glycerol.

### Whole-genome sequencing of ancestral strains and evolved lineages

We used both long- and short-read sequencing to generate high-quality contiguous genomes for the ancestral strains. From a frozen stock, ancestral strains were inoculated in TSB + YE media and incubated for 24 h at 37˚C; 1 mL of the overnight culture was centrifuged at 14,000 × *g* for 10 min to pellet the cells. DNA extraction was performed using the PureLink Microbiome DNA Purification Kit (Invitrogen, Carlsbad, CA) following the manufacturer’s instructions. In addition, 150-bp paired-end Illumina libraries were generated by the Microbial Genome Sequencing Center (Pittsburgh, PA, USA) and sequenced on a NextSeq 2000 sequencer. Low-quality reads were trimmed, and adapter sequences were removed from Illumina sequences using bcl2fastq (version 2.20.0.445). Oxford Nanopore (ONT) PCR-free ligation library preparation was also used to generate long sequence reads. ONT reads were adapter- and quality-trimmed using porechop (36). Hybrid assemblies were generated from Illumina and Oxford Nanopore reads using Unicycler (version 0.4.8) with default settings, and assembly quality was assessed using QUAST (version 5.0.2) (37, 38). Genome annotation was performed with Bakta (version 1.8.1) using the Bakta v5 database (39). To assess genome quality and completeness, we ran BUSCO v5.8.3 on the three ancestral hybrid assemblies using the bacteria_odb_12 data set (40).

We also sequenced the endpoint of the 18 experimentally evolved lineages. Single colonies were isolated for each of the 18 lineages, and DNA was extracted as described above. Whole-genome sequencing of experimentally evolved lineages was conducted with Illumina sequencing only. To investigate allele frequencies within each population, deep sequencing (>15,000,000 reads) was also performed on one population of each BAC-evolved lineage for a total of 9 populations. Each of the 18 experimentally evolved lineages was grown overnight in TSB + YE and normalized to an OD of 1.0 before DNA was extracted using the PureLink Microbiome DNA Purification Kit (Invitrogen; Carlsbad, CA) following the manufacturer’s instructions. We revived cultured strains and populations in TSB + YE (without BAC) prior to DNA extraction to avoid subjecting the populations to an additional generation of strong BAC-mediated selection immediately before sequencing, which could artificially enrich for high-frequency BAC-tolerant alleles and distort allele frequency estimates.

### Serotype inference of *L. monocytogenes* strains

Using a previously described method, the serotypes for ALE_10, ALE_16, and ALE_20 were inferred bioinformatically (41). We ran PhaME (version 1.0.4) with default settings on the genome assembly of the three ancestral isolates and an additional 44 isolates of known serotypes from a study by Hingston et al. (42, 43) (NCBI Bioproject PRJNA329415). To increase the sample size for *L. monocytogenes* serotype 4c, we added nine publicly available 4c genomes to our analysis, which were identified through the NCBI database (Table S1). *L. monocytogenes* EGD-e (serotype 1/2a) was used as the reference genome. An approximately maximum likelihood tree was constructed from the alignment of 253,255 SNPs using FastTree (version 2.1.11), with default parameters, with 100 bootstrap replicates (44, 45). The lineages and serotypes of each experimental isolate were then inferred based on the phylogenetic placement of the known samples.

### Benzalkonium chloride and sanitizer MIC determination

Strains were cultured in triplicate at 37°C for 24 h in TSB + YE. We determined the MIC of each parental strain using a previously described method (46). Briefly, 200 μL TSB + YE containing increasing concentrations of benzalkonium chloride (BAC) was added to a 96-well plate. The inoculum was prepared by normalizing overnight cultures to an OD_600_ of 1.0 and diluting accordingly to 10^−4^–10^−5^ in order to achieve a final cell concentration of approximately 1 × 10^5^ CFU/mL. Next, 10 μL of this inoculum was added to each well and incubated statically overnight at 37°C. Bacterial growth was quantified by measuring the OD_600_ using an absorbance microplate reader (Biotek Instruments Elx800) at 0 h and 24 h growth. The threshold for viable bacterial growth was set at an OD_600_ value of 0.1, and the lowest concentration of BAC to inhibit growth was then identified as the MIC.

A second MIC assay using BAC was conducted on solid media. Tryptic soy agar + 0.6% yeast extract (TSA + YE) plates containing concentrations of 3, 4, 5, and 6 μg/mL BAC were prepared. Ancestor strains and evolved lineages were grown overnight in TSB + YE and then serially diluted to 10^−7^ before plating onto the BAC plates and grown for 36 h at 37°C. Colony counts and imaging were performed using the Interscience Scan 1200.

Finally, we also measured the MIC of a commercial sanitizer containing BAC and another QAC component. Virex II 256 One-Step Disinfectant Cleaner and Deodorant (Diversey, Inc., Fort Mill, SC) was tested at a range of 0.5–5 PPM using the same microplate method described above. This sanitizer contains 8.704% Didecyl Dimethyl Ammonium Chloride and 8.190% n-Alkyl dimethyl benzyl ammonium chloride.

### Testing the role of efflux pumps in QAC tolerance

Reserpine, a known efflux pump inhibitor (EPI), was introduced using a previously described protocol to investigate the role of efflux in the adaptation to BAC (47). Briefly, 1000 μg/mL reserpine stock was prepared in dimethyl sulfoxide (DMSO) and filter-sterilized. Reserpine was added to microplate wells at a final concentration of 20 μg/mL, and the MIC assay was repeated as described above.

### Bacterial growth curve and fitness trade-off analysis

Overnight cultures of ancestor strains and experimentally evolved lineage cultures were standardized to an OD600 of 1.0, and subsequently diluted 10-fold in sterile PBS to achieve a final inoculum of ~10^4^ CFU/mL. A 5 μL volume of this inoculum was then added in triplicate to a 96-well plate (BioLite 96 Well Multidish, Thermo Scientific) with wells each containing 200 mL of TSB + YE (control), TSB + YE with 2 μg/mL BAC, or TSB + YE with 3 μg/mL BAC. Cultures were then grown for 48 h at room temperature, and growth kinetics were measured using the oCelloScope (Biosense Solutions, Denmark) with the following parameters: auto-illumination, auto-focus, image distance = 4.9 mm, images per repetition = 5, repetitions = 48, and repetition interval = 1 h. Total absorption measurements were recorded using a fixed 505 nm wavelength, and growth kinetic analysis was conducted using the normalized total absorption algorithm.

### Comparative genomic analysis

The genomes of the experimentally evolved lineages serially passaged in TSB + YE and serially passaged to TSB + YE and sublethal concentrations of BAC were resequenced at the endpoint of the experiment and compared to their respective high-quality reference genome to identify mutations. Default parameters were used for all software unless otherwise specified. Tally (version 15.065) was used to deduplicate sequence fragments with the following parameters: --pair-by-offset and --with-quality (48). For adapter trimming of the raw reads, TrimGalore (version 0.3.7) was used with the following parameters: --quality 30, --stringency 1, --gzip, and --length 50 (49, 50). BWA (version 0.7.15) was used to map the evolved lineages to the ancestral reference genome (51). Samtools (version 1.4.1) was then used to sort BAM files (52), and sample names were added to the sorted BAM files using the bamaddrg software (available at https://github.com/ekg/bamaddrg). Joint genotyping of ancestral and evolved genomes was then performed using Freebayes (version 1.3.1) (53). To eliminate low-quality variants, site filtering was performed with VCFtools (version 0.1.14) using the following parameters: --remove-filtered-all --minQ 20 --min-meanDP 50 (54). GATK (version 4.0.6) was used to convert the resulting VCF files into a table format (55). SnpEff databases were then built for each of the three ancestors, and SNP annotation was predicted with SnpEff (version 4.1) (56). We used InterPro to predict protein domains in genes containing mutations (57). NCBI Antimicrobial Resistance Finder (AMRFinder) (version 3.10.14) tool was also used to characterize the antimicrobial profile of each isolate (58).

### Antibiotic MIC determination

To test whether increased tolerance to BAC has any off-target effects on antibiotic tolerance, we measured the MIC of several antibiotics in the ancestral and BAC-evolved lineages. Liofilchem MTS (MIC Test Strips) (Waltham, MA) were used to determine the MIC for the following antibiotics: ampicillin, ciprofloxacin, erythromycin, gentamicin, norfloxacin, penicillin G, tetracycline, and vancomycin. Ancestor, BAC-evolved, and TSB-evolved (control) strains were grown overnight in standard conditions and diluted to an OD_600_ of 1.0 before plating 100 μL onto TSA + YE media. The experiment was conducted in duplicate. An MIC test strip for each antibiotic was then placed on the plate with sterile forceps and incubated for 24 h. The MIC was determined as the point where the ellipse of inhibition intersected the antibiotic gradient strip.

### Assessment of key virulence gene *hly* activity using hemolysis assay

To examine whether increased BAC tolerance altered virulence, we measured hemolytic activity, a major contributor of virulence, in the evolved and ancestral lineages. Hemolytic activity was measured for each ancestor and evolved lineage based on a previously described method with minor alterations (59). Briefly, 1 mL of overnight cultures was centrifuged at 14,000 × *g* for 10 min to pellet the bacteria. The supernatant (crude exosubstance) was then transferred to a new microcentrifuge tube, and the pellet was discarded. The crude exosubstance was diluted 2-fold in PBS in a 96-well plate (Thermo Fisher Scientific, Biolite Microwell Plates), leaving a final volume of 100 μL in each well. Negative control wells were prepared with 100 μL sterile PBS, and positive control wells were prepared with 100 μL PBS + 1% Triton X-100. A sheep blood solution was prepared by washing pooled, defibrinated sheep blood (Carolina Biological, Burlington, NC) in cold PBS by repeated centrifugation at 600 × *g* for 10 min. Washing steps were repeated until the supernatant appeared clear and colorless. The clean red blood cell (RBC) pellet was then diluted in cold PBS + 20 mM cysteine to a 3% RBC suspension; 100 μL of the 3% RBC suspension was then added to each well and mixed by pipetting. The microtiter plate was then incubated at 37°C for 30 min. Absorbance readings were performed using an absorbance plate reader (Biotek Instruments Elx800). The following formula was used to quantify the hemolysis percentage: $hemolysis\;\%=\left(1-\frac{OD_{s}}{OD_{t}}\right)\times 100$, where OD_*s*_ is the difference in optical density at 620 nm between the sample and the positive control well and OD_*t*_ is the difference in optical density at 620 nm between the negative and positive control wells.

### Growth kinetics analysis

Growth kinetics analyses were performed among ancestor and evolved lineages in both normal (TSB + YE) and sanitizer stress media (TSB + YE and various BAC concentrations). For both assays, 200 μL of the media was added to each well. Inoculum was prepared by standardizing overnight cultures to an OD_600_ of 1.0 and adding 20 μL to each well. Cultures were grown for 48 h at room temperature, and growth kinetics were measured using the oCelloScope (Biosense Solutions, Denmark) with the following parameters: auto-illumination, auto-focus, image distance = 4.9 mm, images per repetition = 5, repetitions = 48, and repetition interval = 1 h.

To formally compare growth kinetics between ancestral and BAC-evolved lineages, the following three parameters were extracted from the oCelloScope background-corrected absorption (BCA) time-series data for each replicate: (i) maximum BCA, defined as the highest BCA value recorded across the 48-h time course, (ii) area under the growth curve (AUC), calculated by numerical integration of the BCA-vs-time curve using the trapezoidal rule, and (iii) lag phase duration, defined as the first time point at which BCA exceeded 10% of the maximum BCA value for that replicate. For each ancestral strain, the three independently evolved lineages (e.g., ALE_10.1-bac, ALE_10.2-bac, and ALE_10.3-bac) were treated as biological replicates and compared against the three technical replicates of the ancestral strain. Pairwise comparisons of each growth parameter between ancestral and evolved lineages at each BAC concentration were performed using Welch’s two-sample *t*-test. All analyses were conducted in Python v3.12.3 using the pandas v3.0.2 library for data handling, NumPy v2.4.4 for numerical operations (60), and SciPy v1.17.1 for statistical testing (scipy.stats.ttest_ind) and numerical integration (scipy.integrate.trapezoid) (61). A significance threshold of *P* < 0.05 was applied to all comparisons.

### Biophysical modeling of mutational effects in ptsH

To explore the potential functional impacts of mutations identified in experimentally evolved lineages with increased tolerance to BAC, we performed biophysical modeling of ancestral and mutant versions of several candidate proteins. There are no experimental structures available for ptsH (P0A439: https://www.uniprot.org/uniprotkb/P0A439/entry#structure; *Listeria innocua* serovar 6a); therefore, a structural model was downloaded from the AlphaFold database (https://alphafold.ebi.ac.uk/entry/P0A439). The confidence of residue placement in the AlphaFold model was high throughout (pLDDT > 90); hence, no low-confidence residues needed to be removed. The model was then energetically relaxed using parameters optimized to match experimentally derived values of mutational free energy (62). Twenty iterations were performed, and the lowest energy structure was carried forward for estimating mutational effects. Energetic effects of single mutations were estimated using the ddg_cartesian protocol of Rosetta v3.13, along with flags and options previously benchmarked (63, 64). ΔΔ*G* (ddg) estimates for each mutation tested were averaged over 10 iterations of the ddg_cartesian protocol. The results are reported in terms of Rosetta energy units (REU) of the mutation relative to the “wild-type” amino acid (positive ddg values suggest that a variant decreases the stability of the folded protein by increasing the overall free energy of the structure). REU values have been calibrated to roughly correspond to 1–3 kcal/mol. All protein models were imaged using UCSF Chimera.

### RNA sequencing and transcriptomic analysis

In addition to the genotypic changes incurred during long-term exposure to BAC, we also investigated differences in the transcriptome between the evolved lineages and their ancestors in the presence of BAC stress. Evolved strains ALE_10.3*-bac*, ALE_16.3*-bac*, ALE_20.1*-bac*, and ALE_20.3*-bac* were selected, in triplicate, for RNA sequencing because they contained mutations in genes across multiple experimentally evolved lineages. Each culture was revived from freezer stocks by growing in TSB + YE overnight at 37℃; 100 μL of overnight cultures were then passaged into TSB + YE containing 2 μg/mL BAC (3 μg/mL for ALE_16_0415 and its three evolved lineages) and grown to mid-log phase (10 h). Bacteria were then centrifuged at 14,000 × *g* for 10 min to obtain bacterial pellets, which were flash frozen in dry ice. RNA extraction, Ribo-depletion libraries, and RNA sequencing were performed at SeqCenter (Pittsburgh, PA). Briefly, total RNA for each sample was first treated with DNAse (Invitrogen). Illumina’s Stranded Total RNA Prep Ligation with Ribo-Zero Plus Kit was used for library preparation. Sequencing was performed on a NovaSeq 6000, generating 2 × 51 bp reads. Adapter trimming and demultiplexing were performed using bcl-convert (v. 4.0.3).

Genome indexing and mapping were performed using bwa (v0.7.17) with default parameters (51). Samtools (v1.14) was used to sort and index BAM files (52). Bedtools2 (v2.30.0) was used to extract read counts per transcript from sorted BAM files, generating a total read count matrix (65). We adopted a highly conservative approach for identifying differentially expressed (DE) genes using DEApp, a web-based tool that uses a consensus of three different DE analysis methods: DESeq2 (v.1.40.2), edgeR (v. 3.42.4), and limma-voom (v. 3.56.2) (66–69). Differential expression thresholds were defined using log fold change [log2(FC)] values >1 (upregulated) and <−1 (downregulated). A significance threshold was also defined using the multiple-test-corrected *P*-value: (significance level)/(total number of genes). All subsequent log2(FC) and *P*-values reported are from the limma-voom output, as it was the most stringent of the three approaches.

We conducted principal components analysis (PCA) of normalized read counts in JMP Pro (version 17) to examine how the transcriptome profiles of replicates and lineages are clustered (70). Volcano plots were generated using ggplot2 (version 3.4.1) (71). EggNOG mapper (v.2) was used to functionally annotate DE genes (72). UniProt BLAST and NCBI blastx were also used to with “UniProtKB + Swiss-Prot” and “nr” databases, respectively, to provide additional context to DE genes with missing annotations (73, 74). KEGG Mapper and BlastKOALA annotations were generated for the whole genome and used to identify statistically significant overrepresentations of specific KEGG terms among differentially expressed genes using Fisher’s exact tests in R using an FDR cutoff of 0.01 (75).

## RESULTS

### Whole-genome sequencing

We generated closed hybrid genome assemblies using Illumina short-read and Oxford Nanopore long-read data of the three ancestral genomes to ensure accurate mutation prediction in the evolved lineages. The ALE_10-WT and ALE_20-WT assemblies consisted of two contigs with cumulative assembly sizes of 3.07 Mb and 2.93 Mb, respectively, while the ALE_16-WT assembly consisted of three contigs consisting of 3.17 Mb. BLAST search results using the non-redundant protein sequences database show that the small contig in ALE_10-WT (63,873 kb) and ALE_20-WT (76,010 kb) correspond to plasmids pLIS10 and pj1-0208, respectively. The two small contigs in ALE_16-WT correspond to unnamed plasmids with lengths of 57,534 bp and 91,396 bp. BUSCO completeness scores using the bacteria_odb_12 data set were 100% for all three ancestral hybrid genome assemblies, reflecting high-quality complete genome assemblies (40).

We predicted 3,102, 3,168, and 2,869 genes in the ALE_10-WT, ALE_20-WT, and ALE_16-WT genomes, respectively. Additionally, we screened for antimicrobial resistance genes using AMRFinder Plus (58). All ancestral genomes contained *fosX* (encoding a fosfomycin resistance hydrolase FosX) and *lmo0919lin*/*vgaL* (encoding an antibiotic ABC transporter ATP-binding protein). ALE_10-WT and ALE_16-WT also shared an additional lincomycin resistance gene, *abc-f*, as well as the cadmium resistance repressor gene, *cadC*. We performed a BLAST search of the *bcrABC* GenBank reference sequence (JX023284.1) against the ancestral genome assemblies and confirmed that only ALE_16-WT contained this cassette, which is located on the 91,396 bp plasmid. The BAC resistance genes *qacH* and *emrE* were absent from all isolates.

### Phylogenetic relationship of experimental isolates

**Fig 2:**
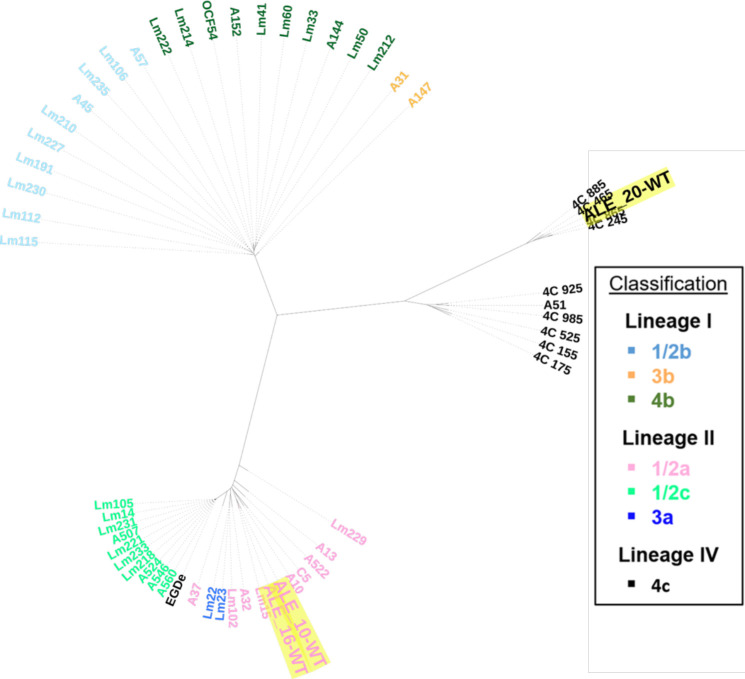
Phylogenetic relationship of the three sequenced ancestral *Li. monocytogenes* strains and 54 *L. monocytogenes* genomes with known serotypes. The phylogeny was inferred by the approximately maximum likelihood approach in FastTree from an alignment of 222,852 single-nucleotide polymorphisms (73). The three currently sequenced genomes were analyzed with 54 previously serotyped *L. monocytogenes* isolates to infer the phylogenetic relationship and serotypes (40). All major splits were supported by bootstrap values of 100%. ALE_10-WT and ALE_16-WT are monophyletic with serotype 1/2a strains, while ALE_20-WT is monophyletic with serotype 4c strains.

We used the PhaME pipeline to identify SNPs in the ALE_10-WT, ALE_16-WT, and ALE_20-WT genomes as well as a diverse collection of 54 assembled *L. monocytogenes* genomes for which serotypes had previously been determined (42). We inferred the phylogenetic relationship of the strains using an alignment of 222,852 SNPs. Our analysis suggests that ALE_10-WT and ALE_16-WT belong to Lineage II, serotype 1/2a, while ALE_20-WT is from Lineage IV, serotype 4c (Fig. 2).

### All BAC-evolved lineages increase tolerance to BAC stress

To model the effects of long-term sublethal exposure to BAC (e.g., in the floor drain of a food processing facility), we grew three lineages of each ancestral strain with daily transfers to fresh media in the presence of (i) control media (TSB + YE) without BAC and (ii) TSB + YE with BAC near lethal concentrations (MIC, 1 μg/mL) for ~70 days (Fig. 1). In the control media, each of the nine lineages reached approximately 840 total generations. Comparatively, lineages grown in media supplemented with BAC grew at about half the rate of the control group, as these nine experimentally evolved lineages reached a range of 370–448 generations.

**Fig 3:**
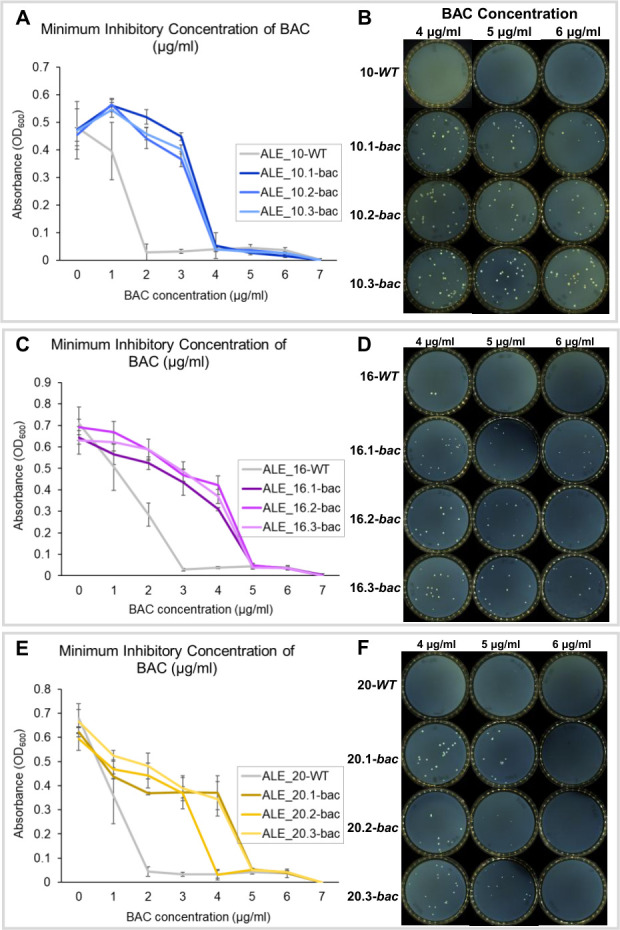
Minimum inhibitory concentration of BAC among BAC-evolved lineages. The MIC of BAC in liquid media was determined by measuring the OD_600_ after 24 h growth in 96-well microtiter plates containing 200 μL of TSB supplemented with increasing concentrations of BAC. The lowest BAC concentration that resulted in bacterial growth measured at OD_600_ < 0.1 was identified as the MIC. The MIC of BAC was also determined on solid TSA supplemented with increasing concentrations (4–6 μg/mL) of BAC for 24 h. Colonies were then quantified using the Interscience Scan 1200. The lowest BAC concentration to exhibit no colonies was deemed the MIC. Liquid and solid media assays were performed among wild-type and BAC-evolved strains (**A and B**) ALE_10*-bac,* (**C and D**) ALE_16*-bac*, and (**E and F**) ALE_20*-bac*.

**Fig 4:**
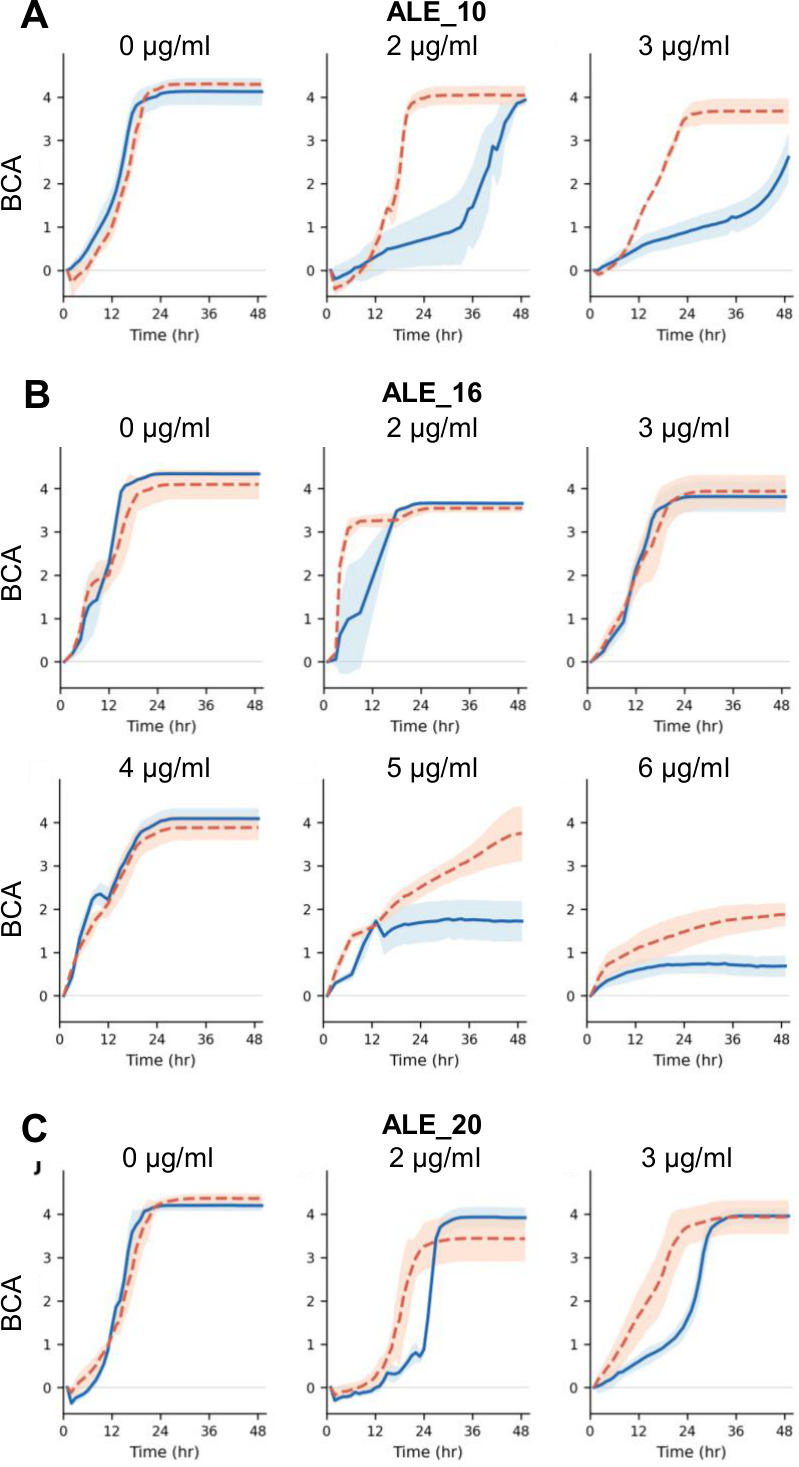
Growth kinetics of ancestral and BAC-evolved *L. monocytogenes* lineages in the absence and presence of BAC stress. Overnight cultures were normalized to an OD600 of 1.0 and subsequently diluted 10-fold in sterile PBS to achieve a final inoculum of ~10⁴ CFU/mL; 5 µL of this inoculum was added in triplicate to a 96-well plate containing media supplemented with BAC concentrations ranging from 0 to 6 µg/mL. Growth kinetics were measured using the oCelloScope over a 48-h period at room temperature and reported as background-corrected absorption (BCA). Each panel shows mean BCA of the ancestral (WT, solid blue line) and BAC-evolved (EVO, dashed red line) lineages across three replicates, with shaded bands representing ±1 standard deviation. Panels **A–C** show ALE_10, ALE_16, and ALE_20, respectively, across BAC concentrations of 0, 2, 3, 4, 5, and 6 µg/mL as indicated. Panels were generated in Python (v3.12.3) using Matplotlib.

Repeated long-term exposure to sublethal concentrations of BAC resulted in increased BAC tolerance for all nine of the experimental replicates in comparison to the ancestral MIC and the MIC of lineages grown in control media lacking BAC (Fig. 3 and 4; Fig. S1). Interestingly, the MIC of BAC differed slightly in liquid and solid media. In liquid media, the MIC of BAC was consistently lower than the MIC in solid media. In liquid media, the MIC for ALE_10_*WT* was 3 μg/mL but increased to 5 μg/mL for all three evolved lineages (Fig. 3), while the MIC in solid media increased from 4 μg/mL to 7 μg/mL (Fig. 3A). Similarly, for ALE_16, the MIC increased from 4 μg/mL to 6 μg/mL in liquid media and increased from 5 μg/mL to 7 μg/mL in solid media. Finally, for ALE_20, the MIC increased from 3 μg/mL to 6 μg/mL (except for strain ALE_20.2-*bac*, which exhibited a MIC of 5 μg/mL) in liquid media and increased from 4 μg/mL to 6 μg/mL on solid media. Despite some variation between liquid and solid media assays, each lineage evolved in the presence of BAC exhibited increased BAC tolerance compared to its ancestor. The MICs for the experimentally evolved lineages in the absence of BAC decreased by 1 μg/mL in ALE_16.1-*tsb*, ALE_16.2-*tsb*, and ALE_20.2-*tsb* relative to the MICs of their respective ancestors, while all other TSB-evolved lineages remained unchanged relative to their ancestor (Fig. S1, Table S2).

To assess whether BAC adaptation was associated with fitness costs in the absence of selective pressure, we compared OD₆₀₀ at 24 and 48 h among ancestral, BAC-evolved, and TSB-evolved lineages grown in TSB + YE without BAC (Fig. S2). BAC-evolved and TSB-evolved lineages were compared using Welch’s *t*-test, with the three independently evolved lineages per group treated as biological replicates. At 24 h, BAC-evolved ALE_16 lineages exhibited significantly higher OD₆₀₀ than TSB-evolved ALE_16 lineages (0.911 ± 0.063 vs. 0.700 ± 0.042; *P* = 0.012), suggesting that BAC adaptation in this *bcrABC*-positive background conferred a general growth advantage independent of BAC stress. No significant differences between BAC-evolved and TSB-evolved lineages were detected for ALE_10 (*P* = 0.239) or ALE_20 (*P* = 0.503) at 24 h. At 48 h, BAC-evolved ALE_10 lineages showed significantly lower OD₆₀₀ than TSB-evolved ALE_10 lineages (0.467 ± 0.011 vs. 0.630 ± 0.046; *P* = 0.021), although this difference was not observed at 24 h, and no BAC-evolved lineage fell below the WT mean at this time point. No significant differences were detected for ALE_16 (*P* = 0.509) or ALE_20 (*P* = 0.598) at 48 h. Taken together, these data reveal no systematic fitness cost associated with BAC adaptation across strain backgrounds or time points.

We also measured the growth kinetics of ancestor and evolved lineages in both control media and in the presence of BAC stress over a 48-h period using the oCelloScope. To formally compare growth performance, maximum BCA, AUC, and lag phase duration were extracted from the BCA time series for each replicate. Overall, BAC-evolved lineages exhibited shorter lag phases and higher BCA values at higher concentrations of BAC compared to the ancestor (Fig. 4).

For ALE_10, BAC-evolved lineages showed significantly higher AUC than the ancestor at both 2 µg/mL (EVO: 131.4 ± 7.3 vs. WT: 57.4 ± 19.9; *P* = 0.024) and 3 µg/mL BAC (EVO: 123.4 ± 5.6 vs. WT: 45.9 ± 10.9; *P* = 0.003), confirming substantially greater cumulative growth under BAC stress. At 0 µg/mL, no significant differences in max BCA or AUC were observed, although lag phase was modestly longer in evolved lineages (8.7 ± 0.5 h vs. 6.0 ± 0.8 h; *P* = 0.025), potentially reflecting a minor fitness cost in the absence of selection. For ALE_16, which uniquely harbors the *bcrABC* benzalkonium chloride resistance cassette conferring elevated baseline BAC tolerance, no significant differences between ancestral and evolved lineages were detected at 0–4 µg/mL BAC, consistent with the ancestor already growing robustly across these concentrations. Significant differences emerged only at concentrations exceeding the ancestral MIC. At 5 µg/mL, evolved lineages showed significantly higher max BCA (EVO: 3.75 ± 0.52 vs. WT: 1.92 ± 0.17; *P* = 0.028) and AUC (EVO: 116.3 ± 7.1 vs. WT: 69.7 ± 14.2; *P* = 0.027), and at 6 µg/mL, max BCA (EVO: 1.89 ± 0.22 vs. WT: 0.75 ± 0.18; *P* = 0.005) and AUC (EVO: 66.6 ± 12.9 vs. WT: 30.0 ± 8.2; *P* = 0.036) were also significantly higher. This pattern is consistent with the MIC data and supports the interpretation that the *bcrABC* cassette likely narrows the selective window for additional adaptation. For ALE_20, no comparisons reached *P* < 0.05; however, biologically meaningful trends were observed at 3 µg/mL BAC, where evolved lineages showed higher (but not statistically significant) AUC (EVO: 138.7 ± 18.0 vs. WT: 105.1 ± 8.1; *P* = 0.103) and a shorter lag phase (EVO: 4.7 ± 1.7 h vs. WT: 9.0 ± 2.2 h; *zP* = 0.093). These comparisons are likely underpowered at *n* = 3, and the observed effect sizes are consistent with the MIC data showing increased BAC tolerance across all three ALE_20-bac lineages.

### Candidate mutations for BAC tolerance in evolved lineages

**Fig 5:**
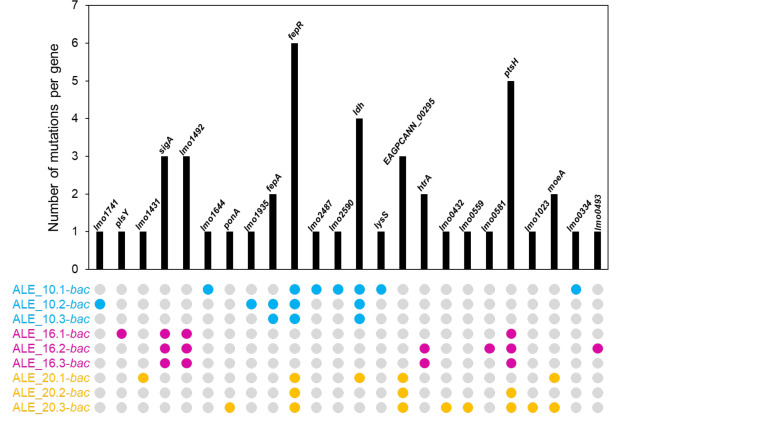
UpSet plot displaying genes containing mutations in BAC-evolved lineages when compared to ancestor strains. Each bar represents a specific gene (common gene name or gene identifier is provided above each bar), and the height of the bar reflects the number of evolved lineages carrying at least one mutation in that gene across the nine experimental lineages, based on comparative genomic analysis with each lineage’s respective ancestor. Colored circles indicate which evolved lineages carry mutations in each gene (colors correspond to ancestral strain identity: blue = ALE_10, pink = ALE_16, yellow = ALE_20), while gray circles indicate the absence of a mutation.

**Fig 6:**
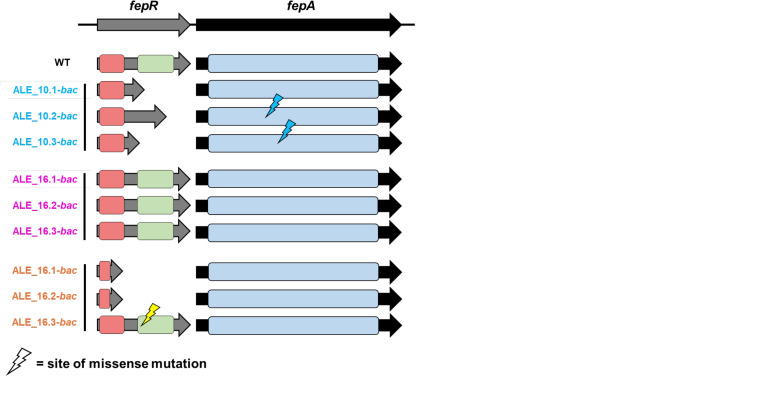
Missense and nonsense mutations observed in the *fepR* and *fepA* genes in ALE_10*-bac* and ALE_20*-bac* lineages. Premature stop codon mutations result in truncated *fepR* proteins in five of six lineages displayed. Blue and yellow lightning bolts indicate the location of missense mutations in ALE_10*-bac* and ALE_20*-bac* lineages, respectively.

We resequenced the genomes of an individual colony per lineage and mapped reads against each lineage’s respective reference genome to identify candidate mutations for BAC tolerance. We observed 26, 17, and 16 mutations in the ALE_10-bac, ALE_16-bac, and ALE_20-bac lineages, respectively. Collectively, mutations were identified within 34 genes (Fig. 5, Table 1). We identified mutations in the same gene in multiple evolved lineages of the same ancestral strain in nine genes (Fig. 5). Three genes (*fepR*, *ldH,* and *ptsH*) contained mutations in evolved lineages from more than one ancestral strain (Fig. 5). No genes showed mutations in experimentally evolved lineages stemming from all three ancestral strains. *fepR*, a negative regulator of the downstream gene *fepA* (fluoroquinolone efflux pump), contained mutations in six of the nine evolved lineages (10.1-*bac*, 10.2-*bac*, 10.3-*bac*, 20.1-*bac*, 20.2-*bac,* and 20.3-*bac*) (Fig. 5 and 6). Five of the six *fepR* mutations were nonsense mutations (including an identical mutation in ALE_20.1-*bac* and ALE_20.2-*bac*), while the other mutation was a missense mutation (Fig. 5 and 6). Additionally, our initial comparative genomic analysis revealed that *fepA* contained missense mutations in ALE_10.2-*bac* and ALE_10.3-*bac* (Fig. 6). Furthermore, deep sequencing revealed an additional missense mutation was present in strain ALE_10.1-*bac* at a lower frequency (57.88%) than the *fepA* mutations identified in the ALE_10.2-*bac* and ALE_10.3-*bac* lineages (Fig. 6; Table 1). This further demonstrates that BAC exposure was a strong selective force, repeatedly driving mutations in both *fepR* and *fepA* across the three ALE_10-bac strains.

**TABLE 1:** List of mutations identified in BAC-evolved strains^*a*^

Isolate	Gene name	Protein product	Gene ID	Mutation type	Mutation description	Mutant frequency
10.1-bac	*oatA*	Peptidoglycan O-acetyltransferase OatA	MEAOLCCM_00079	Frameshift	p.Asn65fs	50.84%
10.1-bac	*mutS*	DNA mismatch repair protein MutS	MEAOLCCM_00197	Frameshift	p.Ile309fs	99.12%
10.1-bac	*lmo1644*	DEAD/DEAH box helicase	MEAOLCCM_00450	Missense	p.Leu645Phe	32.75%
10.1-bac	*lmo1654*	Matrixin family metalloprotease	MEAOLCCM_00461	Frameshift	p.Asn112fs	58.87%
10.2-bac	*lmo1741*	Sensor histidine kinase	MEAOLCCM_00581	Missense	p.His204Tyr	99.79%
10.1-bac, 10.2-bac, 10.3-bac	*purs*	Phosphoribosylformylglycinamidine synthase subunit PurS	MEAOLCCM_00611	Upstream gene variant		63.25% 62.73% 60.80%
10.1-bac	*lmo1855*	D-alanyl-D-alanine carboxypeptidase	MEAOLCCM_00703	Frameshift	p.Pro95fs	47.50%
10.2*-bac*	*lmo1935*	Tyrosine-protein phosphatase	MEAOLCCM_00786	Missense	p.Arg284Leu	99.85%
10.1-bac, 10.2-bac, 10.3-bac	*fepA*	MATE transporter protein FepA	MEAOLCCM_01031	Missense	p.Ala170Val p.Glu151Lys p.Asn182Asp	57.88% 99.77% 96.95%
10.1-bac, 10.2-bac, 10.3-bac	*fepR*	Efflux pump transcriptional regulator FepR	MEAOLCCM_01032	Stop gained	p.Trp100* p.Gln146* p.Glu89*	52.24% 99.68% 97.23%
10.1-bac	*lmo2487*	DUF4097 domain-containing protein	MEAOLCCM_01396	Synonymous	p.Arg237Arg	99.93%
10.1-bac	*lmo2590*	ATP-binding protein	MEAOLCCM_01578	Upstream gene variant		30.92%
10.1-bac	*ecfA1*	ATP-binding protein EcfA1	MEAOLCCM_01590	Missense	p.Val259Ala	29.69%
10.1-bac, 10.2-bac, 10.3-bac	*ldh*	L-lactate dehydrogenase	MEAOLCCM_02068	Missense	p.Ile43Asn, p.Leu148Ile, p.Ala269Thr	33.72% 99.87% 97.25%
10.1-bac	*lysS*	Lysine--tRNA ligase	MEAOLCCM_02090	Missense	p.Met477Ile	99.83%
10.1-bac	*lmo0334*	Flavodoxin-containing protein	MEAOLCCM_02208	Missense	p.Tyr67His	30.14%
10.1-bac	*ltrA*	Low temperature requirement protein A	MEAOLCCM_02265	Upstream gene variant		32.43%
10.3-bac	*dltD*	D-alanyl-lipoteichoic acid biosynthesis protein DltD	MEAOLCCM_02919	Upstream gene variant		89.10%
16.1-bac	*plsY*	Glycerol-3-phosphate acyltransferase	LJHJPIBA_00073	Missense	p.Gly25Asp	99.44%
16.1-bac, 16.2-bac, 16.3-bac	*sigA*	RNA polymerase sigma factor SigA	LJHJPIBA_00250	Missense	p.Ala362Val, p.Ala362Val, p.Arg167Cys	99.64% 65.40% 32.63%
16.1-bac, 16.2-bac, 16.3-bac	*lmo1492*	YqeG family HAD IIIA-type phosphatase^2^	LJHJPIBA_00290	Missense	p.Trp169Leu, p.Ala88Val, p.Arg151Gln	99.34% 100.00% 93.06%
16.1-bac, 16.2-bac, 16.3-bac	unknown	Helix-turn-helix domain-containing protein	LJHJPIBA_01315	Upstream gene variant		99.28% 99.44% 49.62%
16.2-bac, 16.3-bac	*htrA*	Heat-shock protein htrA serine protease	LJHJPIBA_02156	Missense	p.Asn190Asp, p.Val315Glu	99.93% 49.62%
16.2-bac	*lmo0493*	CocE/NonD family hydrolase	LJHJPIBA_02387	Frameshift	p.Tyr75fs	97.33%
16.2-bac	*lmo0581*	Ribosomal RNA large subunit methyltransferase I	LJHJPIBA_02493	Upstream gene variant		98.12%
16.1-bac, 16.2-bac, 16.3-bac	*ptsH*	Phosphocarrier protein HPr	LJHJPIBA_02935	Missense	p.Leu53His	99.63% 99.77% 96.65%
20.1-bac	*ldh*	L-lactate dehydrogenase	EAGPCANN_00195	Missense	p.Asn65Lys	60.96%
20.1-bac, 20.2-bac, 20.3-bac	unknown	Hypothetical protein	EAGPCANN_00295	Synonymous	p.Arg240Arg	99.85% 100% 99.59%
20.3-bac	*lmo0432*	Putative oxidoreductase	EAGPCANN_00441	Synonymous	p.Ser14Ser	99.83%
20.3-bac	*lmo0559*	Magnesium transporter CorA family protein	EAGPCANN_00569	Missense	p.Glu77Lys	100.00%
20.2-bac, 20.3-bac	*ptsH*	Phosphocarrier protein HPr	EAGPCANN_01006	Missense	p.Thr20Asn p.Gly54Val	44.13% 99.73%
20.3-bac	*lmo1023*	Potassium transporter	EAGPCANN_01027	Missense	p.Thr101Met	99.51%
20.1-bac	*moeA*	Molybdopterin molybdenumtransferase	EAGPCANN_01039	Missense	p.Ala32Val	98.88%
20.1-bac	*lmo1431*	ATP-binding protein YbiT	EAGPCANN_01505	Missense	p.Pro311Leu	62.55%
20.3-bac	*infB*	Translation initiation factor IF-2	EAGPCANN_01393	Synonymous	p.Leu438Leu	99.64%
20.3-bac	*ponA*	Penicillin-binding protein 1A	EAGPCANN_02011	Frameshift	p.Tyr755fs	98.00%
20.1-bac, 20.2-bac 20.3-bac	*fepR*	Efflux pump transcriptional regulator FepR	EAGPCANN_02209	Stop gained	p.Ser23* p.Ser23* p.Arg102Gly	62.51% 99.93% 99.51%

^*a*^ Predicted gene annotations were generated from nucleotide sequences using a combination of InterPro, NCBI BLAST, and UniProtKB protein databases. Mutation frequency is defined as the percentage of reads from deep sequencing (>15,000,000 reads) in which the mutation of interest was observed. Missense mutations are annotated as original amino acid, position, and new amino acid (e.g., Leu645Phe = leucine to phenylalanine at position 645); frameshift mutations are annotated with the original amino acid and position followed by "fs" (e.g., Asn65fs = a frameshift beginning at asparagine 65); nonsense (stop gained) mutations are annotated with the original amino acid, position, and an asterisk representing the premature stop codon (e.g., Trp100* = tryptophan-to-stop at position 100). Mutation descriptions are not provided for upstream gene variants because the precise functional consequence of non-coding variants on gene expression is difficult to predict without additional reporter or transcriptomic evidence.

**Fig 7:**
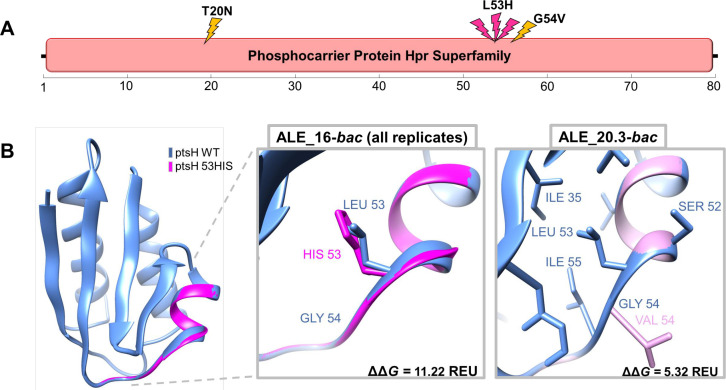
Biophysical modeling of HPr mutations reveals destabilizing effects of BAC-evolved variants. (**A**) Lightning bolts represent mutations in the HPr protein domain within lineages ALE_16-*bac* and ALE_20-*bac*, represented by pink and yellow symbols, respectively. (**B**) Protein models of the HPr proteins in ALE_16-*bac* and ALE_20-*bac* lineages. The wild-type protein is shown in blue, and the destabilized mutant proteins are shown in pink.

We also observed missense mutations in *ptsH*, which encodes an HPr phosphocarrier protein, in five out of nine BAC-evolved lineages (ALE_16.1-*bac*, ALE_16.2-*bac*, ALE_16.3-*bac*, ALE_20.2-*bac*, and ALE_20.3-*bac*) (Fig. 5 and 7, Table 1). Interestingly, the identical mutation NZ_CP090054.1:c.158T > A was observed in all three ALE_16-*bac* lineages in the HPr serine phosphorylation site, resulting in Leu53His. Additionally, ALE_20.2-*bac* and ALE_20.3-*bac* also contained missense mutations in the *ptsH* gene, with the mutation in ALE_20.3-*bac* occurring in the neighboring amino acid as the 16-*bac* lineages, resulting in Gly54Val. Like the mutations observed in ALE_16-*bac* lineages, this mutation also falls within the HPr serine phosphorylation site. Interestingly, the mutation observed in ALE_20.2-*bac* occurs in the HPr-Histine phosphorylation site, resulting in Thr20Asn.

We also observed four instances of missense mutations in the lactate dehydrogenase-encoding gene *ldh1* in the Lactate/malate_DH_N domain (ALE_10.1-*bac*, ALE_10.2-*bac*, ALE_10.3-*bac,* and ALE_20.1-*bac*) (Fig. 5, Table 1). However, we also observed mutations in *ldh* in the ALE_16.2-*tsb-* and ALE_16.3-*tsb*-negative control lineages. These results suggest that alterations to *ldh* could be the byproduct of selective pressure from the media (TSB + YE), rather than from BAC. In the TSB-evolved lineages, we identified 18 mutations in 14 genes. In addition to *ldh*, only two of the genes in which mutations were observed in TSB-evolved lineages overlapped with the mutations identified in the BAC-evolved lineages (Table S2). Mutations observed in both BAC-evolved and TSB + YE-evolved lineages (most notably recurrent missense mutations in *ldh*) were considered likely artifacts of media adaptation rather than BAC-specific selection and were excluded from further analysis.

### Analysis of candidate genes *fepR* and *ptsH*

**Fig 8:**
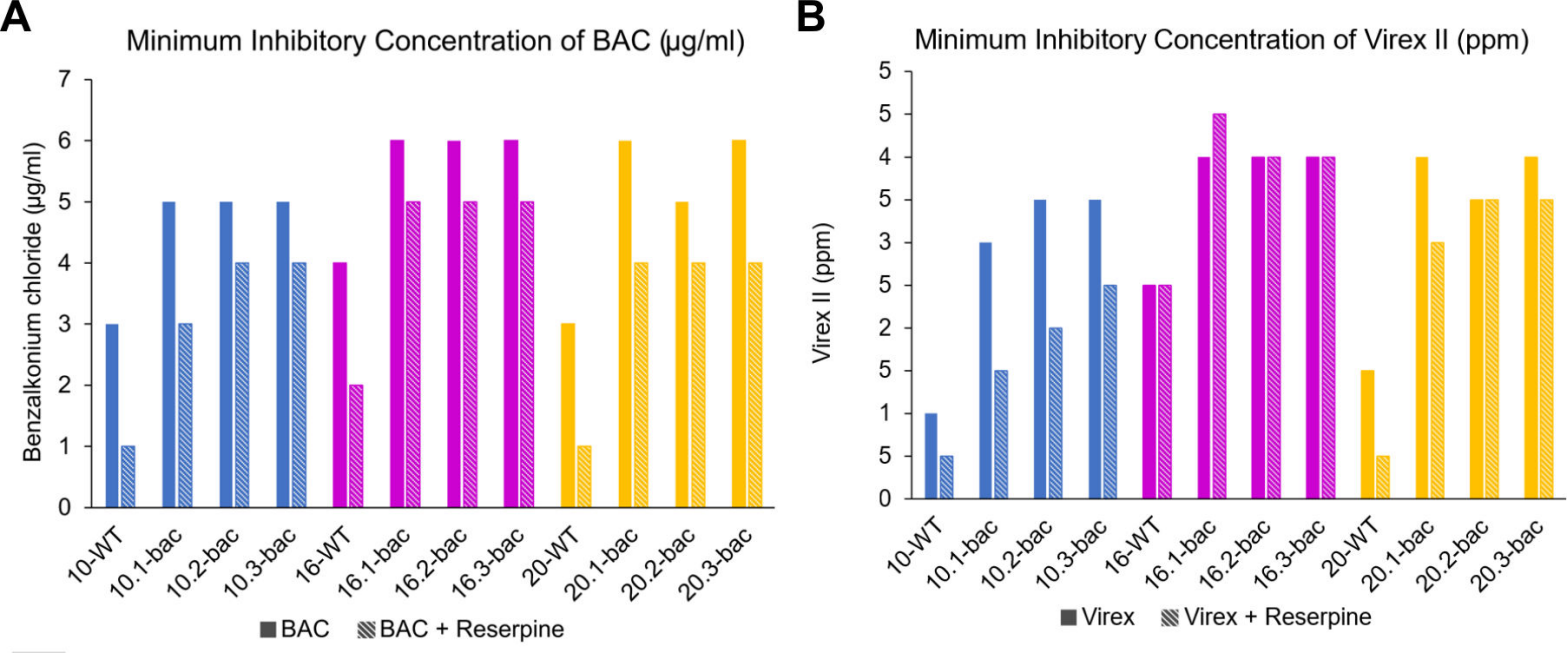
Effect of efflux pump inhibition on BAC and commercial sanitizer tolerance in ancestral and BAC-evolved *L. monocytogenes* lineages. MICs of (**A**) benzalkonium chloride (BAC, µg/mL) and (**B**) Virex II 256 One-Step Disinfectant Cleaner (ppm) were determined for ancestral (WT) and BAC-evolved lineages of ALE_10 (blue), ALE_16 (magenta), and ALE_20 (yellow). MICs were measured by broth microdilution after 24 h growth in 96-well microtiter plates containing 200 µL TSB+YE supplemented with increasing concentrations of each sanitizer; the lowest concentration inhibiting visible growth (OD₆₀₀ < 0.1) was defined as the MIC. Assays were repeated in the presence of reserpine (20 µg/mL), a known efflux pump inhibitor (hatched bars), to assess the contribution of active efflux to sanitizer tolerance. Virex II 256 contains 8.704% didecyl dimethyl ammonium chloride and 8.190% n-alkyl dimethyl benzyl ammonium chloride as active ingredients. Reduced MICs in the presence of reserpine indicate efflux-dependent tolerance.

We further analyzed *fepR* and *ptsH,* as these genes contained mutations in multiple BAC-evolved lineages across different ancestors (Fig. 5), highlighting the likely importance of *fepR* and *ptsH* in BAC tolerance. As noted, *fepR* contained mutations in all lineages of 10-*bac* and 16-*bac*, most of which were nonsense mutations. Because *fepR* negatively regulates the efflux pump *fepA*, we hypothesized that upregulation of *fepA* could lead to increased BAC tolerance, as has been demonstrated previously (25, 76, 77). To test whether *fepA*, and potentially other efflux pumps, drives the increased tolerance to BAC, we grew strains in the presence of the efflux pump inhibitor reserpine. Lineages were grown in the presence and absence of reserpine in media supplemented with BAC and Virex II (which uses BAC and another QAC as active ingredients). The addition of reserpine led to a decrease in the MIC of BAC for all nine BAC-evolved lineages and each ancestor (Fig. 8A). Similarly, in the presence of Virex II, we observed a clear decrease in MIC for both the ancestor and evolved lineages of ALE_10 when reserpine was added (Fig. 8B). For ALE_16, the MIC of the ancestor and BAC-evolved lineages did not change in the presence of reserpine. Finally, in LM_20, the ancestral MIC decreased from 1.5 ppm to 0.5 ppm in the presence of reserpine, while the evolved lineages decreased from 4 ppm to 3 ppm in 20.1-*bac*, from 4 to 3.5 in 20.3-*bac*, and did not change in 20.2-*bac* (3.5 ppm).

HPr was further analyzed because it contained missense mutations in the same protein domains across multiple BAC-evolved lineages. The HPr protein, encoded by *ptsH*, plays a pivotal role in regulating the uptake and phosphorylation of sugars via the phosphotransferase (PTS) system. HPr is also an important cofactor to CcpA, a catabolite control protein that has been shown to regulate gene expression during stress (78–80). Biophysical modeling of the BAC-evolved mutational effect on HPr stability revealed that all five mutations observed in HPr were destabilizing (Fig. 7). The L53H mutation was the most destabilizing (ΔΔ*G* = 11.22 Rosetta Energy Units (REU) and equivalent to approximately 4–5 kcal/mol). This relatively dramatic reduction in predicted protein stability suggests that mutations in the region may only be tolerated under more severe environmental challenges. Convergent evolution of the L53H mutation in the ALE_16-*bac* lineages suggests that this mutation conferred a strong selective advantage during BAC exposure. Furthermore, the occurrence of a mutation at the adjacent residue, G54V, in lineage ALE_20.3-*bac* reinforces a consistent pattern of BAC-induced mutations within this region of the protein domain across two phylogenetically distinct strains.

### BAC-evolved lineages show increased tolerance to a QAC-based sanitizer

All experimentally evolved lineages had higher MICs to Virex II than the ancestor (Fig. 8B), indicating that adaptation under BAC selection increases tolerance to this commercial QAC formulation. Because Virex II 256 contains BAC as well as didecyl dimethyl ammonium chloride (DDAC), these data could reflect direct tolerance to BAC, cross-tolerance to DDAC, or additive effects of the two compounds.

### Some BAC-evolved lineages show resistance to fluoroquinolones

**Fig 9:**
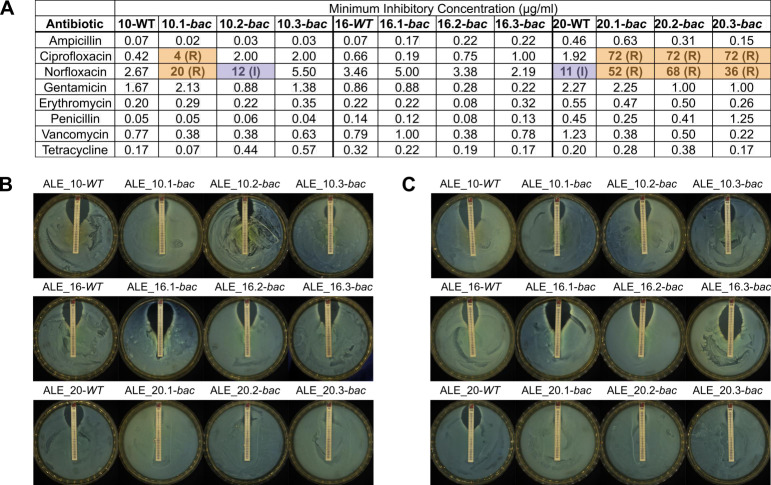
Susceptibility of BAC-evolved lineages to antibiotics. (**A**) Minimum inhibitory concentrations of eight different antibiotics were quantified using Liofilchem Antibiotic strips. Isolates with intermediate antibiotic resistance (I) are indicated with a purple highlighted cell. Isolates with full antibiotic resistance (R) are indicated with an orange highlighted cell based on Staphylococcus spp. CLSI breakpoint values. Images displaying differential zones of inhibition of (**B**) ciprofloxacin and (**C**) norfloxacin antibiotic strips between WT and BAC-evolved lineages.

Previous studies in QAC-resistant strains of *L. monocytogenes* suggest cross-resistance to certain antibiotics (24, 25, 81, 82). To test whether this was the case with our BAC-evolved lineages, we measured the MIC of ampicillin, ciprofloxacin, erythromycin, gentamicin, norfloxacin, penicillin G, tetracycline, and vancomycin in the ancestral and evolved lineages. All evolved lineages of ALE_20-*bac* were highly resistant to the fluoroquinolones ciprofloxacin and norfloxacin and grew at concentrations as high as 32 and 64 μg/mL, respectively (Fig. 9). For comparison, the established MIC breakpoints of ciprofloxacin and norfloxacin for *Staphylococcus spp.* are ≥ 4 µg/mL and ≥ 16 µg/mL, respectively. ALE_10.1-*bac* exhibited resistance to both ciprofloxacin and norfloxacin (MIC = 6 and 20, respectively), while ALE_10.2-*bac* and ALE_10.3-*bac* lineages only had intermediate levels of resistance to the fluoroquinolones (Fig. 9). Interestingly, all evolved lineages of ALE_16-*bac* were susceptible to both ciprofloxacin and norfloxacin despite exhibiting higher tolerance to BAC, further demonstrating the different evolutionary mechanisms between this ancestral strain. To confirm that fluoroquinolone cross-resistance was specific to BAC selection rather than a general consequence of serial passaging, fluoroquinolone MICs were also measured in TSB-evolved control lineages (Fig. S3). All TSB-evolved lineages remained susceptible to both ciprofloxacin and norfloxacin, with MIC values comparable to their respective ancestors, confirming that the elevated fluoroquinolone MICs observed in ALE_10-bac and ALE_20-bac lineages are attributable to BAC-driven selection rather than to general adaptation during serial passaging.

### Hemolytic activity in BAC-evolved lineages is decreased compared to ancestor

A hemolysis assay was conducted using sheep blood to quantify the activity of listeriolysin O, a hemolysin encoded by the key virulence factor *hly*. Expression of *hly* is used as a proxy for the activity of the main *Listeria* virulence regulon, PrfA; hence, the usefulness of the hemolysis assay as a measure of virulence in *L. monocytogenes* (79). All nine BAC-evolved lineages showed a decrease in hemolytic activity compared to their respective ancestors (Fig. S4), with statistically significant reductions observed in all three ALE_20-bac lineages. To determine whether this reduction was specific to BAC adaptation or a more general consequence of serial passaging, hemolytic activity was also assessed in TSB-evolved control lineages (Fig. S5). ALE_16 and ALE_20 TSB-evolved lineages showed no significant differences from their ancestors, suggesting that the reduction in hemolytic activity in the corresponding BAC-evolved lineages is attributable to BAC-specific selective pressure rather than general adaptation to the growth medium. However, two ALE_10 TSB-evolved lineages (10.1-tsb and 10.3-tsb) also exhibited significantly reduced hemolytic activity relative to the ALE_10 ancestor (*P* < 0.05), consistent with the pattern of medium-driven adaptation observed at other loci in this background (e.g., *ldh*), and complicating interpretation of the hemolysis reduction observed in ALE_10-bac lineages specifically.

### Differential gene expression analysis between ancestor and evolved lineages in the presence of BAC

**Fig 10:**
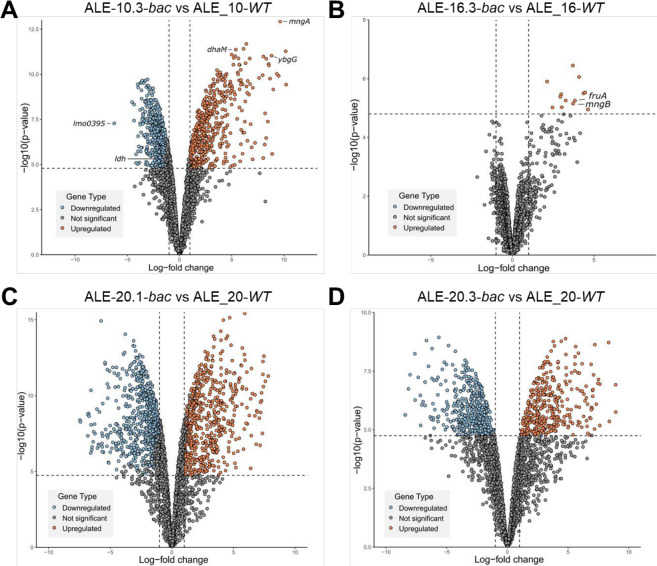
Volcano plots show differential gene expression of BAC-evolved lineages relative to their respective parental strains. Volcano plots for lineages (**A**) ALE_10.3-bac, (**B**) ALE_16.3-bac, (**C**) ALE_20.1-bac, and (**D**) ALE_20.3-bac. Vertical dashed lines represent differential expression thresholds, which were defined using log fold change [log2(FC)] values >1 (upregulated) and <−1 (downregulated). The horizontal dashed line represents the significance threshold defined using a Bonferroni-corrected *P*-value.

We performed RNA sequencing of each ancestral strain as well as ALE_10.3-*bac*, ALE_16.3-*bac*, ALE_20.1-*bac*, and ALE_20.3-*bac* in biological triplicate during BAC stress (growth in TSB + YE containing 2 μg/mL BAC for 10 h) (mid-log phase) to examine transcriptome profile differences across samples. These lineages were selected as they encompassed lineages with mutations in *fepR, fepA,* and *ptsH* (Fig. 10, Table 1).

We performed principal component analysis (PCA) of DESeq2-normalized read counts (Fig. S6) to determine the relationship between transcriptome profiles across samples. Across all three samples, ancestral replicates clustered independently from the BAC-adapted replicates, suggesting that the transcriptomic profiles vary considerably between ancestral and BAC-adapted lineages. 20-*WT*, ALE_20.1-*bac*, and ALE_20.3-*bac* replicates all clustered independently of one another, which suggests that the independent evolution of two lineages arising from the same ancestor led to significant variation in their transcriptomic profiles. Based on our significance thresholds, ALE_10.3*-bac* had 483 upregulated and 573 downregulated genes, ALE_16.3*-bac* had 13 upregulated and zero downregulated genes, ALE_20.1*-bac* had 613 upregulated and 656 downregulated, and ALE_20.3*-bac* had 354 upregulated and 447 downregulated genes (Fig. 10). ALE_20.1*-bac* and 20.3*-bac* share 295 upregulated genes and 349 downregulated genes.

We performed KEGG term enrichment analysis to identify associations between specific functional categories in the differentially expressed gene sets (Table 2; Table S4 through S6). We found that among genes downregulated in the presence of BAC, the functional category “Genetic Information Processing” was significantly overrepresented in ALE_10.3-*bac* (*P* = 7.0E−9), ALE_20.1-*bac* (*P* = 1.56E−17), and ALE_20.3-*bac* (*P* = 4.75E−14) (Table S6). Additionally, carbohydrate metabolism genes were overrepresented among genes upregulated in the presence of BAC across all four BAC-evolved lineages used in this analysis (ALE_10.3-*bac: P* = 5.28E−23, ALE_16.3-*bac: P* = 0.009, ALE_20.1-*bac: P* = 3.52E−11, and ALE_20.3-*bac: P* = 1.4E−4) (Table S4). KEGG pathway enrichment analysis revealed that several function subcategories of carbohydrate metabolism were significantly associated with upregulation, most notably being genes of the phosphotransferase system (PTS) (Table 2). The PTS system was significantly overrepresented among genes upregulated in ALE_10.3-*bac* (*P* = 6.41E−10), ALE_16.3-*bac* (*P* = 7.51E−4), ALE_20.1-*bac* (*P* = 6.38E−9), and ALE_20.3-*bac* (*P* = 3.34E−5) (Table 2). Specifically, PTS genes *dhaK, dhaM, dhaL, gmuB, manZ, and ulaA* were all upregulated in two or more of the 4 BAC-evolved lineages selected for RNA-seq (Fig. 10). Another PTS gene, *ptsH,* in which missense mutations were observed in all three lineages of ALE_16-*bac*, ALE_20.2-*bac*, and ALE_20.3-*bac,* was downregulated in ALE_20.1-*bac* and ALE_20.3-*bac* and showed no significant differential expression in other lineages.

**TABLE 2:** KEGG term enrichment of upregulated genes^*a*^

ALE lineage	Description	KEGG term	Odds ratio	*P*-value	FDR *P*-value
10.3-bac	Fructose and mannose metabolism	00051	15.37	1.00E−10	2.02E−08
	Phosphotransferase system (PTS)	02060	10.76	6.41E−10	6.48E−08
	Carbon metabolism	01200	3.37	1.92E−05	1.29E−03
	Citrate cycle (TCA cycle)	00020	18.26	3.59E−05	1.45E−03
	Starch and sucrose metabolism	00500	6.86	3.57E−05	1.45E−03
	Microbial metabolism in diverse environments	01120	2.39	9.19E−05	3.09E−03
16.3-bac	Starch and sucrose metabolism	00500	50.81	4.86E−06	9.87E−04
20.1-bac	PTS	02060	9.2	6.38E−09	1.28E−06
	Fructose and mannose metabolism	00051	6.6	2.13E−05	1.42E−03
	Starch and sucrose metabolism	00500	7.83	1.73E−05	1.42E−03
	Lipoic acid metabolism	00785	10.26	7.29E−05	3.38E−03
	Carbon metabolism	01200	3.09	8.44E−05	3.38E−03
	Carbon fixation in photosynthetic organisms	00710	15.94	1.61E−04	5.38E−03
20.3-bac	PTS	02060	5.59	3.34E−05	6.69E−03
	Starch and sucrose metabolism	00500	7.52	6.75E−05	6.73E−03
	Sulfur relay system	04122	16.62	1.01E−04	6.73E−03

^*a*^ The strain identifier, KEGG term description, KEGG term ID, odds ratio (the ratio of the odds of observing the KEGG term associated with differentially expressed genes relative to the odds of observing the KEGG term associated with non-differentially expressed genes), and *P*-value (Fisher’s exact test with significance threshold set to *P*-value < 0.01) are provided for all significantly enriched KEGG terms in the upregulated gene sets.

The efflux pump *fepA* and its negative regulator *fepR* were also differentially expressed. *fepA* was upregulated in ALE_20.1-*bac* and ALE_20.3-*bac* but downregulated in ALE_10.3-*bac* (Fig. 10). Interestingly, *fepR* was upregulated in both ALE_20.1-*bac* and ALE_20.3-*bac*, although these lineages contain a premature stop codon mutation and a missense mutation, respectively, likely leading to upregulation of *fepA*.

We looked closely at virulence genes because we observed a trend of reduced virulence, based on our hemolysis assay in evolved lineages. For instance, the hemolysin-producing gene *hly* was downregulated in ALE_10.3-bac (LFC = −2.39, *P*-value = 4.91E−8) and ALE_20.3-bac (LFC = −2.56, *P*-value = 6.41E−6). Phospholipase-encoding genes *plcA* and *plcB* were downregulated in both ALE_20.1-*bac* and ALE_20.3-*bac*, but *plcA* was upregulated in ALE_10.3-bac. Finally, *inlB*, which encodes an internalin protein that is key to *Listeria* virulence, was upregulated in ALE_10.3-*bac* (LFC = 3.86, *P*-value = 1.04E−10), ALE_20.1-*bac* (LFC = 1.31, *P*-value = 1.06E−09), and ALE_20.3-*bac* (LFC = 4.44, *P*-value = 1.69E−09).

### Discussion

We experimentally evolved nine *L. monocytogenes* lineages, stemming from three distinct ancestral strains, in the presence of BAC-stress for 70 days (~400 generations) to examine their routes of adaptation. Through comparative genomic analysis of the endpoint lineages, we identified 51 total mutations among 34 different genes. Several genes, including *fepR, fepA,* and *ptsH,* were identified as potential determinants of BAC adaptation due to multiple observations of mutations in those genes across multiple evolved lineages.

Several other studies have revealed that mutations in *fepR* caused a significant increase in QAC tolerance. In the wild-type *L. monocytogenes*, *fepR* is a negative transcriptional regulator of *fepA*, a MATE-family efflux transporter (21). However, the proposed mechanism of action for increased tolerance among *fepR* mutants is that the mutation results in a truncated or non-fuctional FepR protein, which results in the upregulation of the efflux pump (25, 83). Previous studies have provided support for this mechanism, with results showing that in the presence of reserpine, a known efflux pump inhibitor, the sanitizer-adapted phenotype is lost, and tolerance returns to WT levels (21, 23, 24). These results demonstrate that the *fepA* efflux pump gene and its transcriptional repressor, *fepR*, are at least partly responsible for the adaptation to BAC in ALE_10 and ALE_20 BAC-evolved lineages. Our results show that the *fepR* premature stop codon mutations evolved independently in five different strains, which provides strong evidence that this genotype is in part responsible for the increase in BAC tolerance (Fig. 6). Repeated independent premature stop codons were also observed in another study that tested the effects of QAC stress in *L. monocytogenes* using short-term (10 days) experimental evolution (25). To validate the role of *fepR* in BAC tolerance, we also tested the effect of reserpine on QAC tolerance. The addition of reserpine did decrease the MICs in the ALE_10-*bac* and ALE_20-*bac* lineages, although this decrease did not return to WT MIC levels as reported in another study (Fig. 8A) (21). Missense mutations observed in *fepA* of lineages ALE_10.2-*bac* and ALE_10.3-*bac*, or other lineage-specific mutations, could also potentially contribute to an additive tolerance to BAC. Interestingly, no mutations were observed in the *fepR/fepA* mechanism among the three ALE_16-*bac* lineages despite a marked increase in BAC tolerance with respect to the 16-*WT* strain. We predict that the *fepA/fepR* mechanism was unchanged in the three 16-*bac* lineages due to this strain uniquely harboring a known benzalkonium chloride resistance cassette, *bcrABC*. While the absence of *fepR* mutations in ALE_16-bac lineages is consistent with the hypothesis that pre-existing *bcrABC* activity reduces selective pressure on the *fepA/fepR* axis, we acknowledge that this interpretation is based on a single *bcrABC*-positive strain. Future work testing multiple independent *bcrABC*-positive backgrounds or directly comparing isogenic strains with and without the cassette would strengthen this conclusion. The *bcrABC* cassette is predicted to be a potential source of persistent phenotypes in the food processing environment, as it confers increased tolerance to QACs, and it is known to be transferable to other species. The *brcABC* cassette is normally carried on the plasmid pLM80, first identified in *L. monocytogenes* H7550, a strain that was responsible for a multistate outbreak in 1998–1999, which resulted in 8 deaths and over 50 illnesses (18, 84). This example highlights the importance of genetic background in selection favoring different mutations, as ALE_10 and ALE_16 are closely related but differ in the presence of the *bcrABC* cassette.

The introduction of an EPI provided further evidence of varying tolerance pathways as the BAC MICs of all three ALE_16-*bac* lineages remained the same in the presence of reserpine, while the MICs of ALE_10-*bac* and ALE_20*-bac* lineages decreased in the presence of reserpine (Fig. 8). This is consistent with previous findings in which the BAC MICs of two *bcrABC-*positive strains were unchanged in the presence of reserpine, while 12 out of 15 *bcrABC*-negative strains exhibited decreased MICs in the presence of reserpine (85). Five bcrABC-positive strains exhibited MICs of BAC as high as 12 μg/mL both in the presence and absence of reserpine, while reserpine treatment resulted in decreased MICs for all 18 bcrABC-negative strains (16).

Previous studies have found that bacterial adaptation to BAC stress is correlated with resistance to fluoroquinolone antibiotics. In *L. monocytogenes* specifically, the *fepR/fepA* mechanism has previously been described in association with fluoroquinolone resistance, which is consistent with the results of the present study (Fig. 9) (76, 86). Interestingly, ALE_16-*bac* lineages did not contain mutations in *fepR* or *fepA* and did not demonstrate any significant increase in the MICs of either ciprofloxacin or norfloxacin. This provides further evidence that the *fepR/fepA* mechanism that confers increased BAC tolerance is also the main determinant of fluoroquinolone resistance. It should be noted that lineages 10-*WT* and 16-*WT* are both from serotype 1/2a of *L. monocytogenes* lineage II, whereas 20-*WT* is distantly related and belongs to serotype 4c of lineage IV (Fig. 2). This is a significant finding, as it shows convergent evolution (*fepR/fepA*) across two phylogenetically distinct strains of *L. monocytogenes*.

Other non-specific efflux pump genes have been described in *L. monocytogenes* and associated with tolerance to QACs and other biocides (87, 88). Several studies have explored a mechanism involving a multidrug resistance efflux pump encoded by gene *mdrL* (**m**ulti-**d**rug **r**esistance ***L**isteria*), which is regulated by transcriptional repressor *ladR* (87, 88). Huillet (88) constructed a *ladR* null mutant and showed that LadR binds to and regulates the MdrL efflux pump under standard conditions, but the repressor is inactive during rhodamine stress. Studies have also shown that the gene *lde* (***L****isteria* **d**rug **e**fflux) encodes another efflux pump that contributes significantly to fluoroquinolone resistance, although the results vary regarding the gene’s involvement in BAC stress response (89, 90). This suggests that *L. monocytogenes* utilizes multiple efflux pump repressor mechanisms similar to the *fepR/fepA* genes described above.

The recurrent mutations observed in the *ptsH* may represent a potentially novel and compelling mechanism of sanitizer tolerance in *L. monocytogenes. ptsH* is particularly interesting because a recurrent mutation resulting in an L53H substitution emerged in all three 16-*bac* lineages, and one of the two mutations observed in *ptsH* in the 20-*bac* lineages resulted in a substitution in the neighboring amino acid residue (G54V) (Fig. 7). The emergence of mutations in *ptsH* in close proximity across lineages from different starting strains suggests strong selection. This observation provides evidence of evolutionary convergence on a trait between lineages 16-*bac* and 20-*bac.* Our protein modeling results suggest that these mutations are highly destabilizing to the phosphocarrier protein HPr, but the relationship between this protein and BAC is unclear. Future work should directly test whether *ptsH* mutations alone are sufficient to confer increased BAC tolerance, for example, by introducing individual substitutions (e.g., L53H, G54V, and T20N) into isogenic wild-type backgrounds via allelic exchange and measuring the resulting MIC shift.

We also conducted RNA sequencing of ancestral strains and several BAC-evolved lineages under BAC stress. The expression profiles of the sanitizer-evolved lineages provide additional context to the transcriptome response to BAC tolerance. Genes involved in carbohydrate metabolism and, more specifically, the PTS system were significantly associated with genes upregulated under BAC stress (Table S4). These findings, in conjunction with the recurrent mutations found in *ptsH*, led us to hypothesize that upregulation and modulation of the PTS system is a key, efflux-independent determinant of BAC tolerance in *L. monocytogenes.* Previous studies have described an association between PTS genes and QAC tolerance, although further research is needed to understand the precise mechanisms of action of the PTS proteins under QAC stress (46, 91). The phosphocarrier protein HPr, encoded by *ptsH*, has been found to play a role in regulating antibiotic resistance in combination with the CroR/CroS signaling system in *Enterococcus faecalis* (92). This study described a physical association between HPr and CroR, a transcriptional regulator involved in cephalosporin resistance. The association between these two proteins reveals an interface in which HPr restricts CroR-dependent cephalosporin resistance. Further mutations of HPr led to a disruption of the HPr-CroR protein association and increased cephalosporin resistance without losing HPr functionality. While it is unclear exactly how the HPr mutations influence BAC tolerance in our system, it is conceivable that a mechanism similar to HPr-CroR could be at play in *L. monocytogenes,* where HPr provides nutritional control of some unknown, CroR-like, transcriptional regulator that contributes to antimicrobial resistance. Taken together, our experimental model to study the evolution of BAC tolerance validated previous findings that revealed the role of *fepR* and *fepA* and revealed new insights into the role of *ptsH* and, in general, the PTS system. Additional studies are required to understand how lineage-specific mutations may contribute to BAC tolerance.

## Data Availability

*L. monocytogenes* wild-type strains FSL-N1-304 (ALE_10), FSL-N1-334 (ALE_16), and FSL-J1-158 (ALE_20) whole-genome Illumina and Oxford Nanopore Technologies sequencing data are available through the NCBI SRA run accession numbers SRR17006605 and SRR17067427, respectively. The reference genome assemblies for wild-type strains FSL-N1-304 (ALE_10), FSL-N1-334 (ALE_16), and FSL-J1-158 (ALE_20) are available through GenBank accession numbers GCA_021391455.1, GCA_021403065.1, and GCA_021391475.1, respectively. Illumina whole-genome sequencing data for BAC-evolved lineages are available through NCBI SRA run accession numbers SRR21641536 and SRR21641535, respectively. RNA-sequencing data are available through NCBI BioProject PRJNA782601.

## SUPPLEMENTAL MATERIAL

Supplemental material msystems.01809-25-s0001.pdf Figures S1-S6 and Tables S1-S6.

ASM does not own the copyrights to Supplemental Material that may be linked to, or accessed through, an article. The authors have granted ASM a non-exclusive, world-wide license to publish the Supplemental Material files. Please contact the corresponding author directly for reuse.
