# Supplementary material for: Experimental evolution reveals genetic and transcriptomic mechanisms underlying benzalkonium chloride tolerance and fluoroquinolone cross-resistance in Listeria monocytogenes
Source: mSystems. 2026 Jul 24;11(8):e01809-25. doi: 10.1128/msystems.01809-25 (PMC13483377; doi:10.1128/msystems.01809-25)
Supplement: Supplementary file 1 [file msystems.01809-25-s0001.pdf]

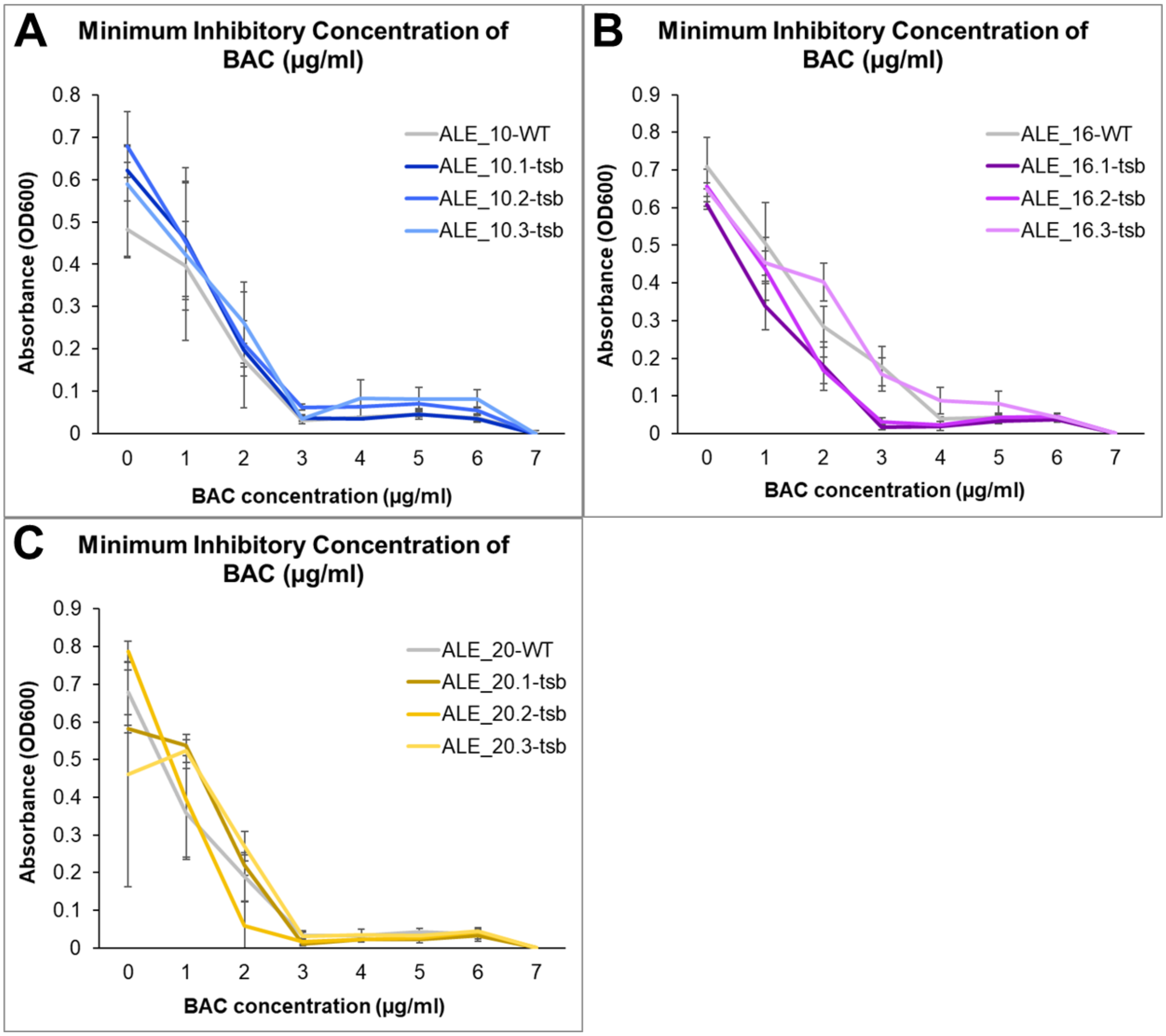

Figure S1. Minimum inhibitory concentration of BAC among TSB-evolved lineages. The MIC of BAC was determined for wild type and TSB-evolved lineages by measuring the OD_{600} after growth for 24 hours in 96-well microtiter plates containing 200 µl volumes of TSB supplemented with increasing concentrations of BAC. The lowest BAC concentration where bacterial growth measured $OD_{600} < 0.1$ was determined as the MIC.

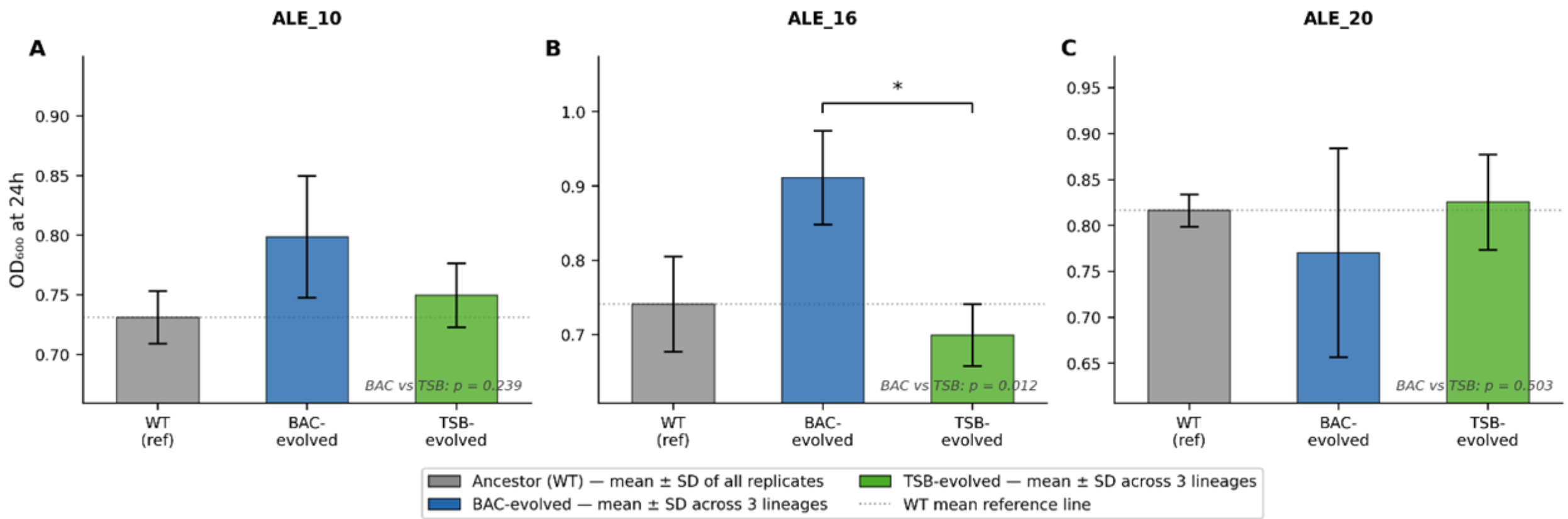

Figure S2. Growth of ancestral, BAC-evolved, and TSB-evolved *L. monocytogenes* lineages in the absence of BAC stress. OD_{600} was measured after 24 hours and in TSB+YE without BAC supplementation for ancestral (WT, gray), BAC-evolved (blue), and TSB-evolved (green) lineages of ALE_10, ALE_16, and ALE_20 (A-C, respectively). Bars represent mean OD_{600} ± SD. For WT, the mean and SD are calculated across all technical replicates (n = 3 at 24 h; n = 6 at 48 h). For BAC-evolved and TSB-evolved groups, the mean and SD are calculated across the three independently evolved lineages, each represented by the mean of its technical replicates. The dotted horizontal line indicates the WT mean for reference. Significance brackets indicate pairwise comparisons between BAC-evolved and TSB-evolved groups assessed by Welch's two-sample t-test. * = $p < 0.05$.

A

	Minimum Inhibitory Concentration (µg/ml)											
**Antibiotic**	**10-WT**	**10.1-*tsb***	**10.2-*tsb***	**10.3-*tsb***	**16-*WT***	**16.1-*tsb***	**16.2-*tsb***	**16.3-*tsb***	**20-WT**	**20.1-*tsb***	**20.2-*tsb***	**20.3-*tsb***
Ciprofloxacin	0.42	0.30	0.57	0.44	0.66	0.32	0.50	0.50	1.92	1.50	1.75	1.13
Norfloxacin	2.67	2.25	1.88	3.00	3.46	1.75	1.00	1.50	11 (I)	4.50	9.50	4.50

B

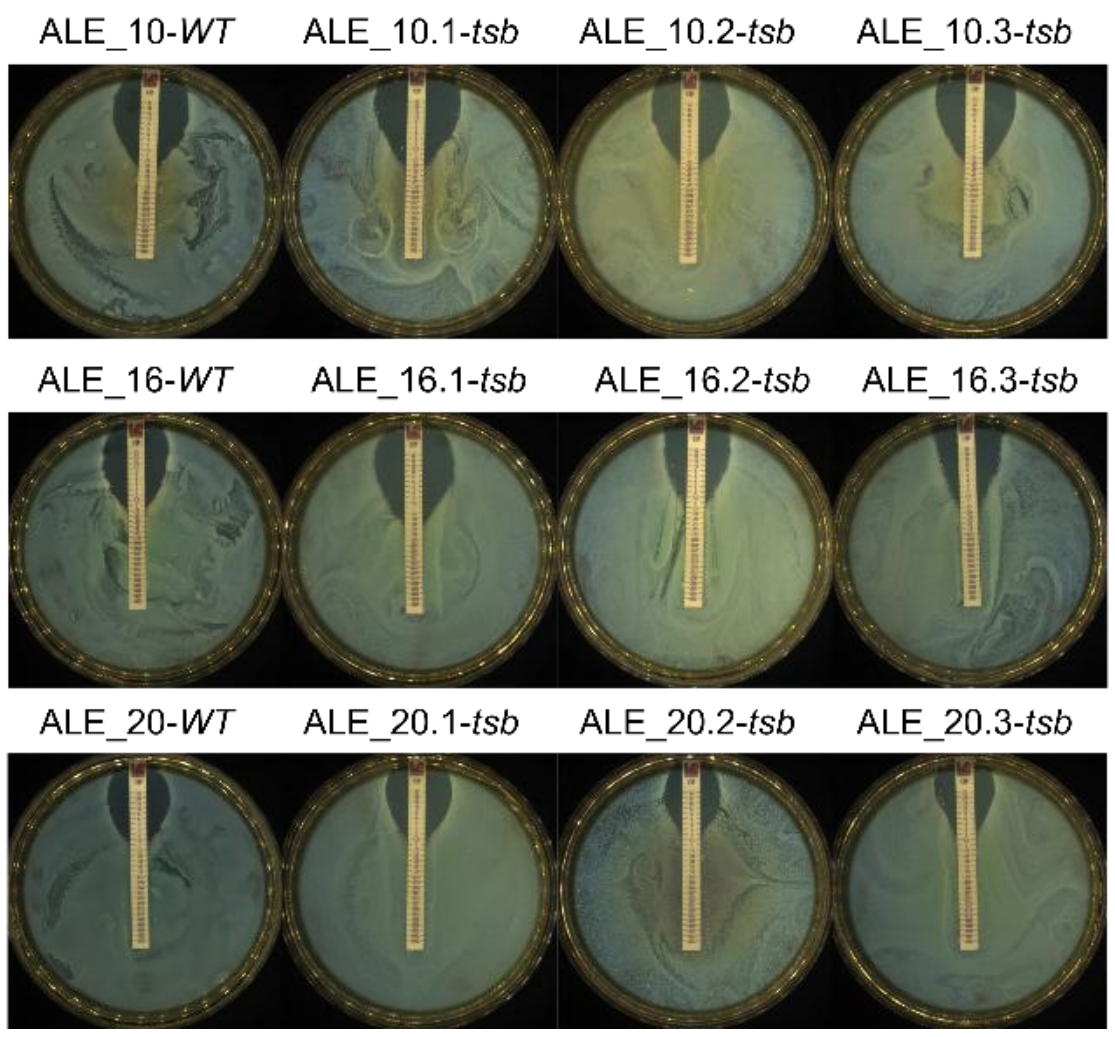

C

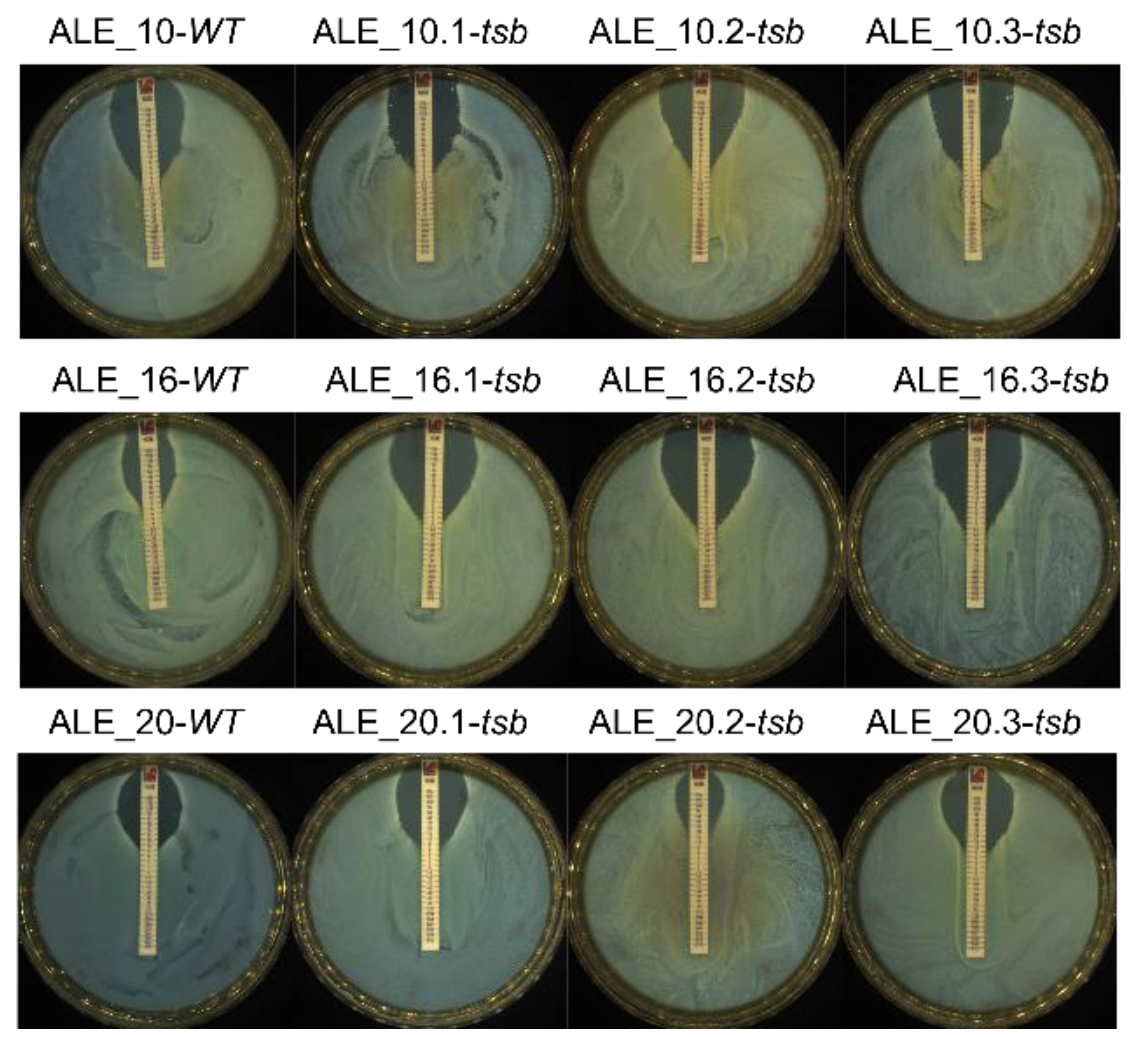

Figure S3. Susceptibility of TSB-evolved lineages to antibiotics. (A) Minimum inhibitory concentrations of fluoroquinolone antibiotics were quantified for TSB-evolved strains using Liofilchem Antibiotic strips. Images displaying zones of inhibition from **B)** ciprofloxacin and **C)** norfloxacin antibiotic strips between WT and TSB-evolved lineages. Isolates with intermediate antibiotic resistance (I) are indicated by a purple highlighted cell.

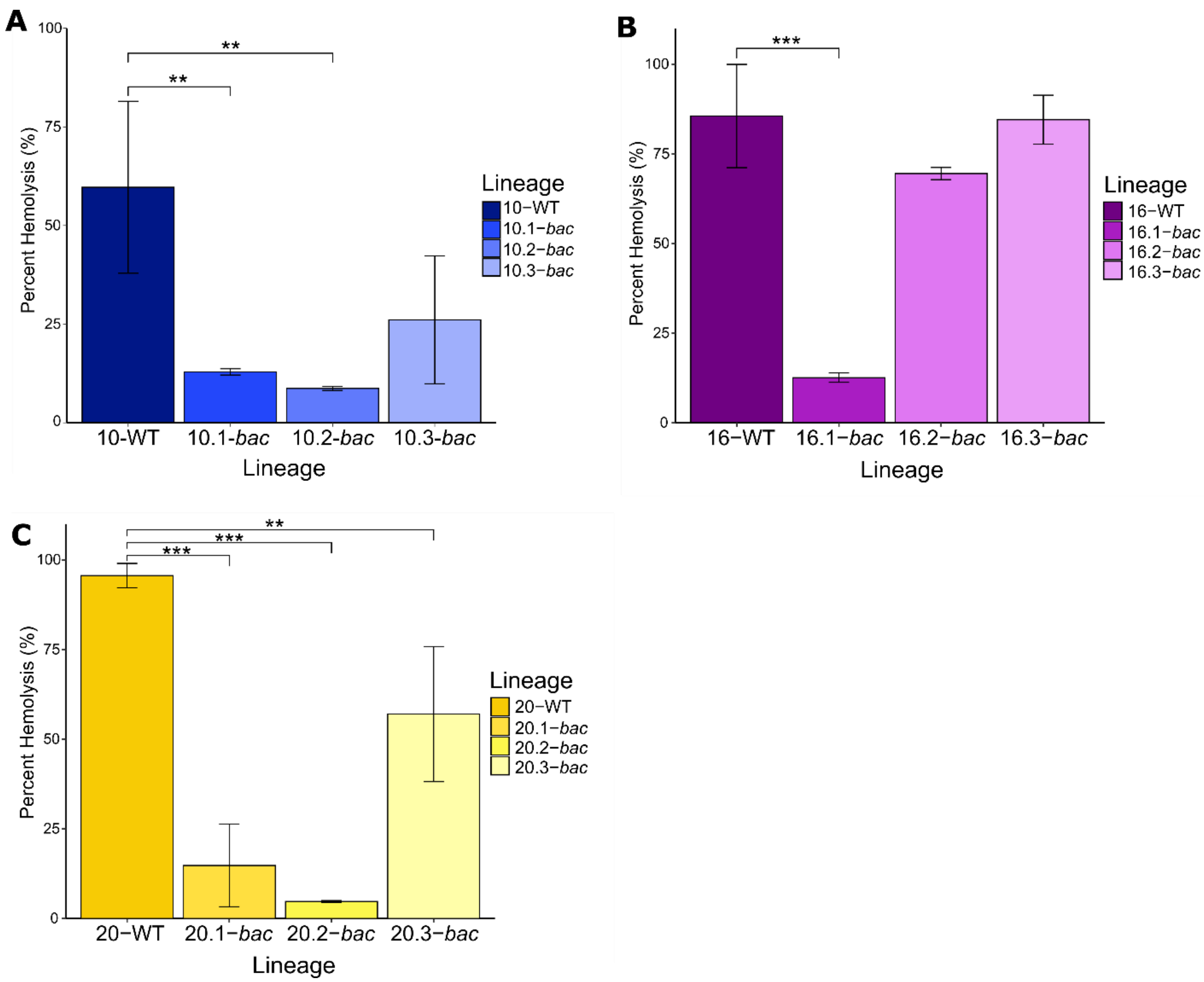

Figure S4. Comparison of hemolytic activity between WT and BAC-evolved lineages. Activity of the key virulence gene, *hly*, was quantified by using defibrinated sheep blood to assess the impact of BAC adaptation on *L. monocytogenes* virulence. Percent hemolysis results are displayed above for **A)** ALE_10, **B)** ALE_16, and **C)** ALE_20 lineages. A double asterisk (**) indicates p-value < 0.05 and triple asterisk (***) indicates a p-value < 0.001.

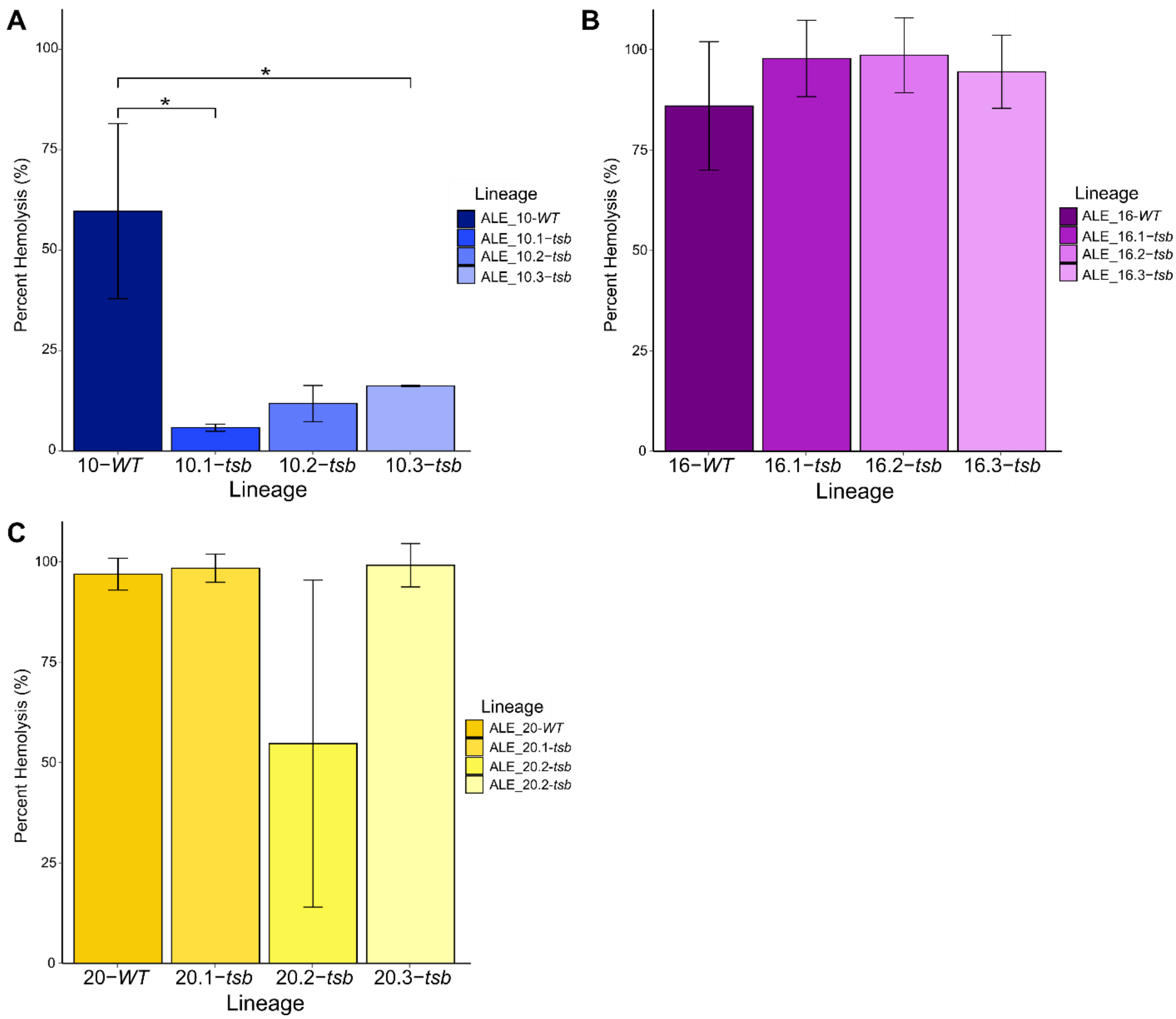

Figure S5. Comparison of hemolytic activity between WT and TSB-evolved lineages. Activity of the key virulence gene, *hly*, was quantified using defibrinated sheep blood in TSB-evolved control lineages to assess whether serial passage in TSB+YE media alone altered *L. monocytogenes* virulence independent of BAC selection. Percent hemolysis results are displayed for A) ALE_10, B) ALE_16, and C) ALE_20 TSB-evolved lineages. Each bar represents the mean of three biological replicates ± standard deviation. Statistical comparisons were performed using a one-way ANOVA followed by Dunnett's post-hoc test, with the respective ancestral (WT) strain serving as the control group. A double asterisk (**) indicates $p < 0.05$ and a triple asterisk (***) indicates $p < 0.001$ relative to the WT ancestor.

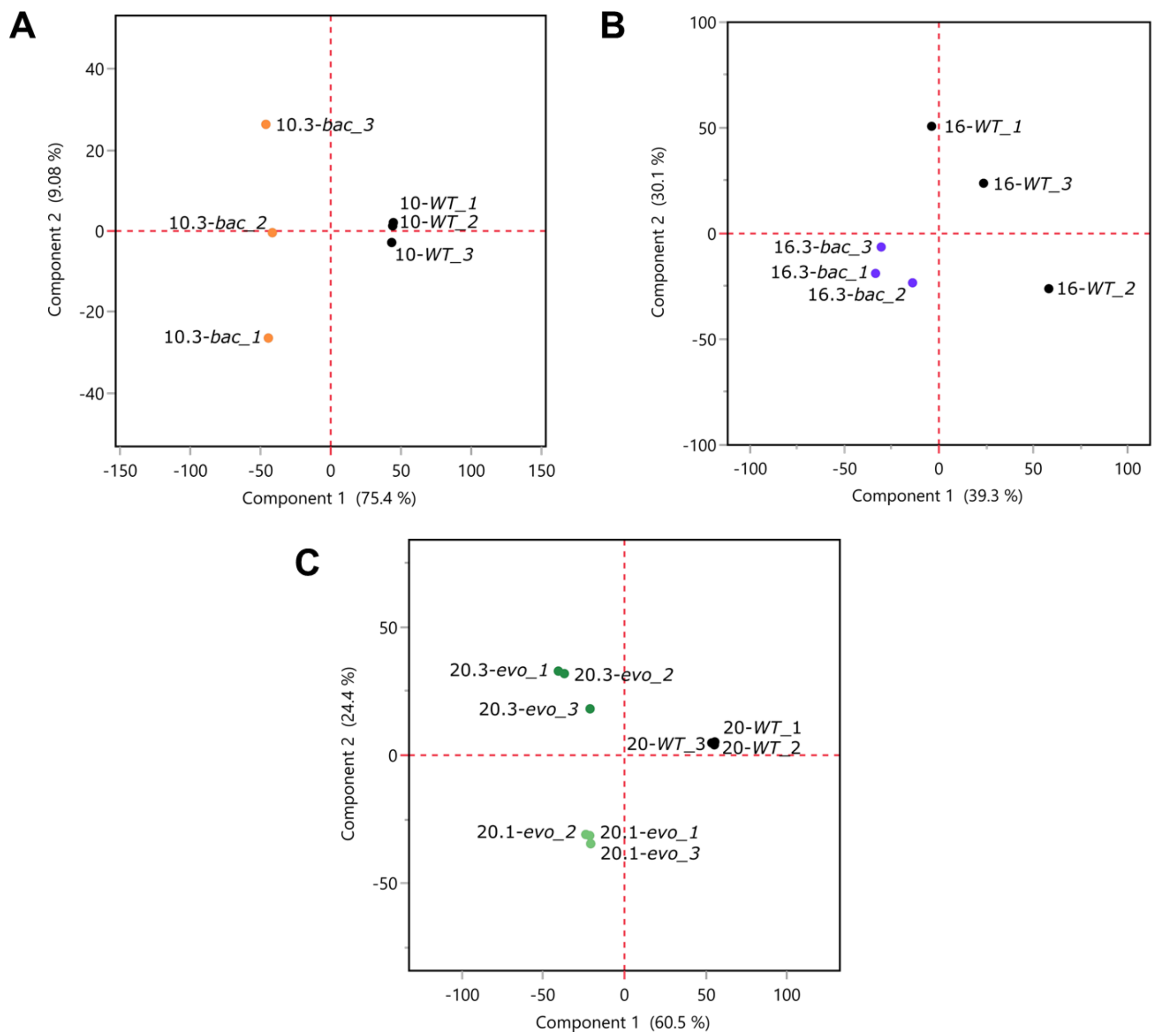

Figure S6. Principal component analysis of RNA-sequencing gene expression data during BAC stress reveals distinct clustering of the ancestor and BAC-evolved strains. Normalized read counts were generated using DESeq2, and PCA was performed in JMP. **A)** 10.3-bac replicates are displayed using orange dots, **B)** 16.3-bac replicates are displayed using purple dots, and **C)** 20.1-bac and 20.3-bac replicates are displayed using light and dark green dots, respectively. All ancestral replicates are represented with black dots.

Table S1. Accession numbers for each publicly available genome used in our phylogenetic analysis. The identifiers in the first column correspond to the identifiers used in Figure 2.

Strain Name	Genbank ID	Assembly ID
A10	GCA_001709845	ASM170984
A13	GCA_001709895	ASM170989
A144	GCA_001709885	ASM170988
A147	GCA_001710805	ASM171080
A152	GCA_001710095	ASM171009
A31	GCA_001710015	ASM171001
A32	GCA_001710385	ASM171038
A37	GCA_001711565	ASM171156
A45	GCA_001711505	ASM171150
A507	GCA_001711645	ASM171164
A51	GCA_001710115	ASM171011
A522	GCA_001710245	ASM171024
A524	GCA_001711915	ASM171191
A546	GCA_001711835	ASM171183
A560	GCA_001712295	ASM171229
A57	GCA_001712305	ASM171230
C5	GCA_001710705	ASM171070
Lm102	GCA_001709375	ASM170937
Lm105	GCA_001709365	ASM170936
Lm106	GCA_001709455	ASM170945
Lm112	GCA_001712135	ASM171213
Lm115	GCA_001712155	ASM171215
Lm14	GCA_001709545	ASM170954
Lm15	GCA_001709565	ASM170956
Lm191	GCA_001710785	ASM171078
Lm210	GCA_001710365	ASM171036
Lm212	GCA_001710715	ASM171071
Lm214	GCA_001710455	ASM171045
Lm218	GCA_001709645	ASM170964
Lm22	GCA_001709765	ASM170976
Lm221	GCA_001711245	ASM171124
Lm222	GCA_001712375	ASM171237
Lm227	GCA_001711015	ASM171101
Lm229	GCA_001709555	ASM170955
Lm23	GCA_001708675	ASM170867

Lm230	GCA_001710845	ASM171084
Lm231	GCA_001709625	ASM170962
Lm233	GCA_001709465	ASM170946
Lm235	GCA_001709605	ASM170960
Lm33	GCA_001710535	ASM171053
Lm41	GCA_001711385	ASM171138
Lm50	GCA_001710445	ASM171044
Lm60	GCA_001710905	ASM171090
OCF54	GCA_001711145	ASM171114

Table S2. Benzalkonium chloride minimum inhibitory concentrations of ancestral, BAC-evolved, and TSB-evolved *L. monocytogenes* lineages.

Ancestral Strain	Lineage	Evolution Treatment	BAC MIC (µg/mL)
ALE_10	WT	Ancestor	3
ALE_10	10.1-bac	BAC-evolved	5
ALE_10	10.2-bac	BAC-evolved	5
ALE_10	10.3-bac	BAC-evolved	5
ALE_10	10.1-tsb	TSB-evolved	3
ALE_10	10.2-tsb	TSB-evolved	3
ALE_10	10.3-tsb	TSB-evolved	3
ALE_16	WT	Ancestor	4
ALE_16	16.1-bac	BAC-evolved	6
ALE_16	16.2-bac	BAC-evolved	6
ALE_16	16.3-bac	BAC-evolved	6
ALE_16	16.1-tsb	TSB-evolved	3
ALE_16	16.2-tsb	TSB-evolved	3
ALE_16	16.3-tsb	TSB-evolved	4
ALE_20	WT	Ancestor	3
ALE_20	20.1-bac	BAC-evolved	6
ALE_20	20.2-bac	BAC-evolved	5
ALE_20	20.3-bac	BAC-evolved	6
ALE_20	20.1-tsb	TSB-evolved	3
ALE_20	20.2-tsb	TSB-evolved	2
ALE_20	20.3-tsb	TSB-evolved	3

Table S3. Mutations identified from comparative genomic analysis of wild type and TSB-evolved strains. Protein products annotations were predicted from a combination of InterPro, NCBI BLAST, and UniProtKB protein databases.

Isolate	Gene ID	Mutation Type	Mutation Description	Gene name	Protein Product
10.1-tsb	MEAOLCCM_00197	frameshift	p.Ile309fs	*mutS*	DNA mismatch repair protein MutS
10.1-tsb	MEAOLCCM_01396	synonymous	p.Arg237Arg	*lmo2487*	hypothetical protein
10.1-tsb	MEAOLCCM_01426	frameshift	p.Thr23fs	*lmo2517*	GNAT family protein
10.1-tsb	MEAOLCCM_01467	UGV		*lmo2557*	lipid kinase
10.3-tsb	MEAOLCCM_01913	synonymous	p.Pro375Pro	*dnaC*	replicative DNA helicase
10.1-tsb	MEAOLCCM_02090	missense	p.Met477Ile	*lysS*	Lysine--tRNA ligase
10.1-tsb	MEAOLCCM_02955	missense	p.Asp36Tyr	*lmo1004*	YdcF family protein
16.2-tsb	LJHJPIBA_01898	frameshift	p.Met237fs	*lmo0050*	sensor histidine kinase
16.2-tsb	LJHJPIBA_02041	missense	p.Thr208Asn	*ldh1*	L-lactate dehydrogenase
16.3-tsb	LJHJPIBA_02041	missense	p.Asp159Asn	*ldh1*	L-lactate dehydrogenase
16.2-tsb	LJHJPIBA_02153	frameshift	p.Lys182fs	*lmo0289*	transcriptional regulator
16.3-tsb	LJHJPIBA_02153	frameshift	p.Phe357fs	*lmo0289*	transcriptional regulator
20.1-tsb	EAGPCANN_00040	synonymous	p.Pro265Pro	*ptcA*	putrescine carbamoyltransferase
20.1-tsb	EAGPCANN_00040	missense	p.Ala324Val	*ptcA*	putrescine carbamoyltransferase
20.1-tsb	EAGPCANN_00056	missense	p.Gly93Arg	*lmo0050*	histidine kinase
20.3-tsb	EAGPCANN_00195	missense	p.Ala269Thr	*ldh1*	L-lactate dehydrogenase
20.1-tsb	EAGPCANN_01039	missense	p.Ala32Val	*lmo1042*	molybdopterin biosynthesis protein MoeA
20.1-tsb	EAGPCANN_01852	frameshift	p.Tyr48fs	*ade*	adenine deaminase
20.1-tsb	EAGPCANN_02594	missense	p.Arg255Gly	*cls*	cardiolipin synthase

Table S4. KEGG term enrichment of downregulated genes during BAC stress. KEGG terms were assigned for the whole genome and enrichment analysis was performed using Fisher's exact test to identify terms that were significantly overrepresented in the BAC-evolved strains (p-value < 0.01).

KEGG Term Enrichment of Downregulated Genes

	**Description**	**KEGG Term**	**Odds Ratio**	**p-value**	**FDR p-value**
**10.3-*bac***	Ribosome	03010	5.68	7.47E-10	1.51E-07
	Aminoacyl-tRNA biosynthesis	00970	5.81	2.05E-05	2.07E-03
**20.1-*bac***	Ribosome	03010	25.89	6.73E-25	1.35E-22
	Flagellar assembly	02040	29.10	3.85E-13	3.85E-11
	Aminoacyl-tRNA biosynthesis	00970	9.71	7.09E-08	4.72E-06
	Bacterial chemotaxis	02030	33.86	6.71E-06	3.35E-04
	ABC transporters	02010	2.76	3.26E-05	1.30E-03
**20.3-*bac***	Ribosome	03010	16.25	1.26E-21	2.52E-19
	Flagellar assembly	02040	10.42	1.36E-08	1.36E-06
	ABC transporters	02010	3.27	4.11E-06	2.74E-04
	Arginine biosynthesis	00220	12.31	2.10E-05	1.05E-03

Table S5. BlastKOALA Enrichment analysis of upregulated genes during BAC stress. Terms were assigned for the whole genome and enrichment analysis was performed using Fisher's exact test to identify terms that were significantly overrepresented in the BAC-evolved strains (p-value < 0.01).

	Description	Odds Ratio	p-value	FDR p-value
**10.3-*bac***	Carbohydrate metabolism	4.59	5.28E-23	1.16E-21
	Unclassified: metabolism	2.95	7.19E-06	7.91E-05
	Genetic Information processing	0.33	1.82E-04	1.34E-03
**16.3-*bac***	Environmental information processing	6.79	8.99E-03	1.03E-01
	Carbohydrate metabolism	5.80	9.35E-03	1.03E-01
**20.1-*bac***	Carbohydrate metabolism	2.91	3.52E-11	7.74E-10
	Human diseases	17.53	2.18E-04	2.40E-03
	Genetic Information processing	0.35	3.68E-04	2.70E-03
	Unclassified: metabolism	2.22	1.72E-03	9.48E-03
**20.3-*bac***	Human diseases	29.23	1.67E-05	3.67E-04
	Carbohydrate metabolism	2.13	1.49E-04	1.64E-03
	Genetic Information processing	0.40	1.21E-02	8.89E-02

Table S6. BlastKOALA enrichment analysis of downregulated genes during BACs stress. Terms were assigned for the whole genome and enrichment analysis was performed using Fisher's exact tests to identify terms that were significantly overrepresented in the BAC evolved strains (p-value < 0.01).

	Description	Odds Ratio	p-value	FDR p-value
**10.3-*bac***	Genetic Information processing	2.83	7.00E-09	1.54E-07
**20.1-*bac***	Genetic Information processing	4.27	1.56E-17	3.42E-16
	Cellular processes	6.06	4.46E-09	4.91E-08
	Protein families: signaling and cellular processes	1.97	1.41E-04	1.03E-03
	Carbohydrate metabolism	0.52	4.10E-04	2.26E-03
	Environmental information processing	1.86	8.00E-04	3.52E-03
**20.3-*bac***	Genetic Information processing	3.96	4.75E-14	1.05E-12
	Cellular processes	5.76	3.45E-08	3.79E-07
	Carbohydrate metabolism	0.27	1.18E-07	8.64E-07
	Environmental information processing	1.97	8.26E-04	4.54E-03
	Unclassified: metabolism	0.23	1.08E-03	4.76E-03
